# Supplementary material for: An integrated organoid omics map extends modeling potential of kidney disease
Source: Nat Commun. 2023 Aug 14;14:4903. doi: 10.1038/s41467-023-39740-7 (PMC10425428; doi:10.1038/s41467-023-39740-7)
Supplement: Supplementary file 1 — Supplementary Information [file 41467_2023_39740_MOESM1_ESM.pdf]

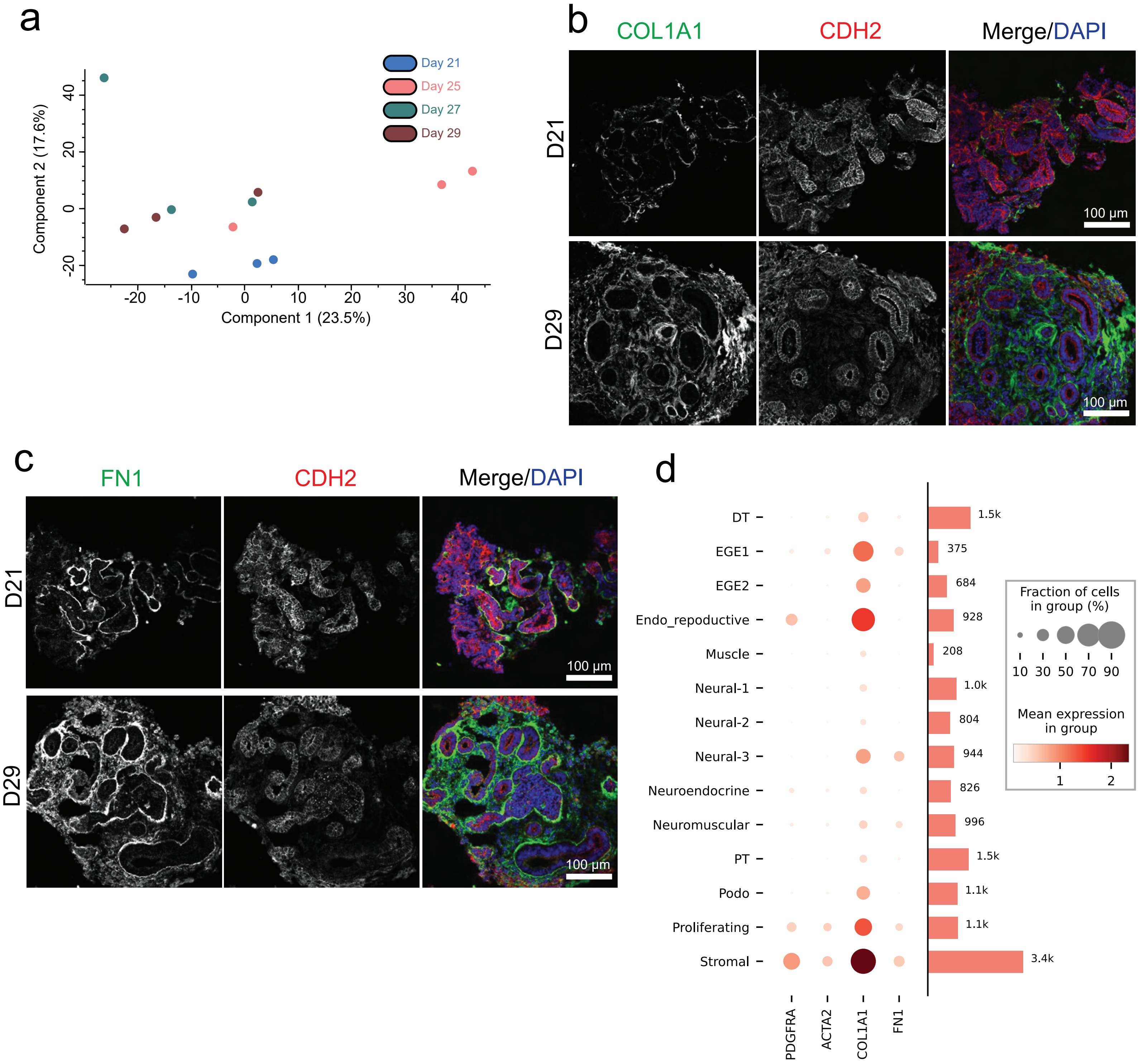

Supplemental Figure 1 | Proteome analysis of maturing kidney organoids. Relevant figures 1, 2.
A) Principal component analysis of organoid proteomes during cell culturing from D21 to D29. B) Immunofluorescence imaging of kidney organoids at D21 (top panel) and D29 (bottom panel) showing expression of proximal tubule marker N-cadherin (CDH2) and extracellular matrix markers (B) collagen type I alpha 1 chain (COL1A1) and (C) fibronectin type I (FN1), n=3, representative images shown, scale bar: 100 µm. D) Dot plot showing transcript expression of PDGFRA, ACTA2, COL1A1 and FN1 in organoid cell types by single cell transcriptional profiling as in Fig. 2C. Bar length and associated numbers indicate the number of cells in the clusters. Source data are provided as a Source Data file.

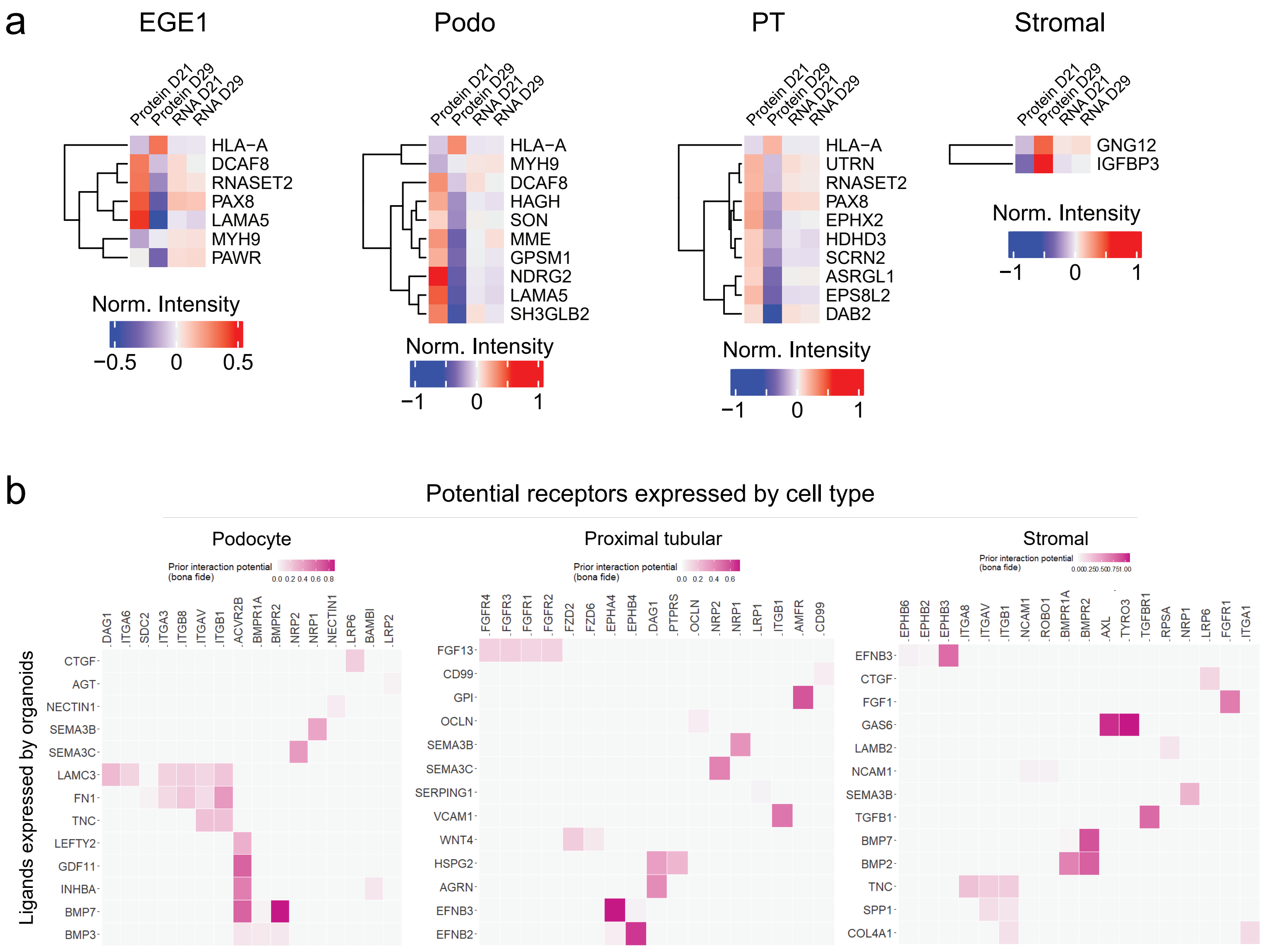

Supplemental Figure 2 | Cell-type specific gene expression and intercellular interaction in organoids. A) Heatmap clustering of bulk RNA and bulk protein of transcript cell-type markers for early glomerular epithelial 1 (EGE1), maturing podocyte (Podo), proximal tubular (PT) and stromal cells. The top differentially expressed proteins D29 versus D21 (FDR<0.01) which showed little regulation on the transcript level were plotted. B) NicheNet analysis predicting ligand-target interactions in maturing organoid cell types, incorporating evidence of target engagement (expression of predicted target genes) based on proteins differentially expressed D29 versus D21 and assigned to cell types (Suppl. Tables 2,4) as in Fig. 2D. Source data are provided as a Source Data file.

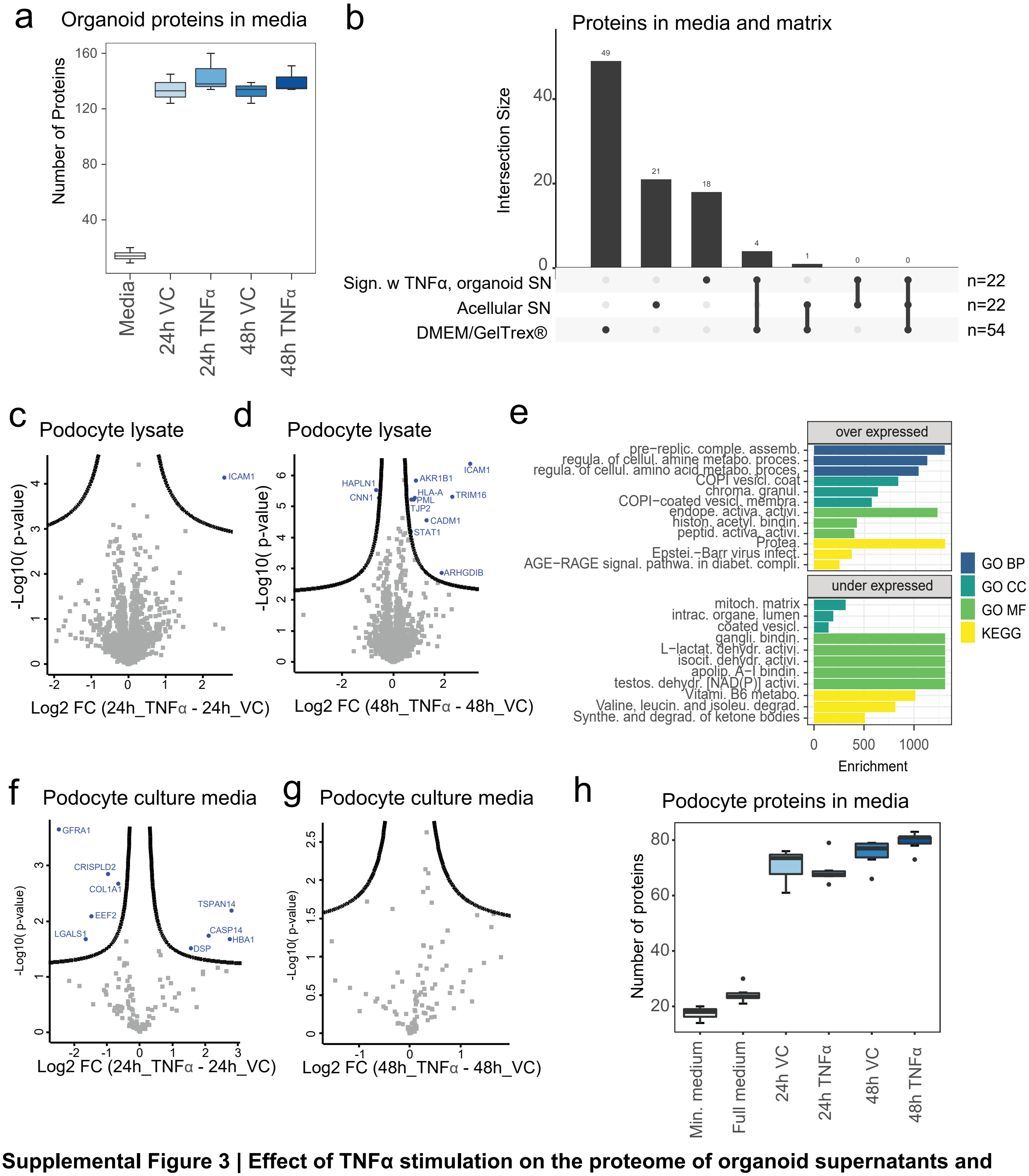

Supplemental Figure 3 | Effect of TNFα stimulation on the proteome of organoid supernatants and cultured podocytes.

A) Total proteins detected in VC- and TNFα-treated organoid culture media, as compared to media alone without organoids (n=3). Plots: median, boxes represent the interquartile range (IQR, 25-75% percentile), whiskers represent the largest/smallest values within 1.5 x IQR. B) Contribution of proteins to the secretome from TNFα-stimulated organoids, acellular wells or combination of media/matrix. C) Differential proteomics expression analysis (log2 fold change) of cultured podocyte cells (C,D) and overlying culture medium (F,G) following treatment with TNFα for 24h or 48h, compared to VC. Two-sided t-test with an FDR<0.2 for supernatants and an FDR<0.1 for cells. E) Associated Gene Ontology (GO) term enrichment plots relative to (C,D). The GO-term panels correspond to over- and under-expressed proteins. H) Cultured podocytes secreted ~70 detectable proteins on average into the medium (n=6). Plots: median, boxes represent the interquartile range (IQR, 25-75% percentile), whiskers represent the largest/smallest values within 1.5 x IQR. Source data are provided as a Source Data file.

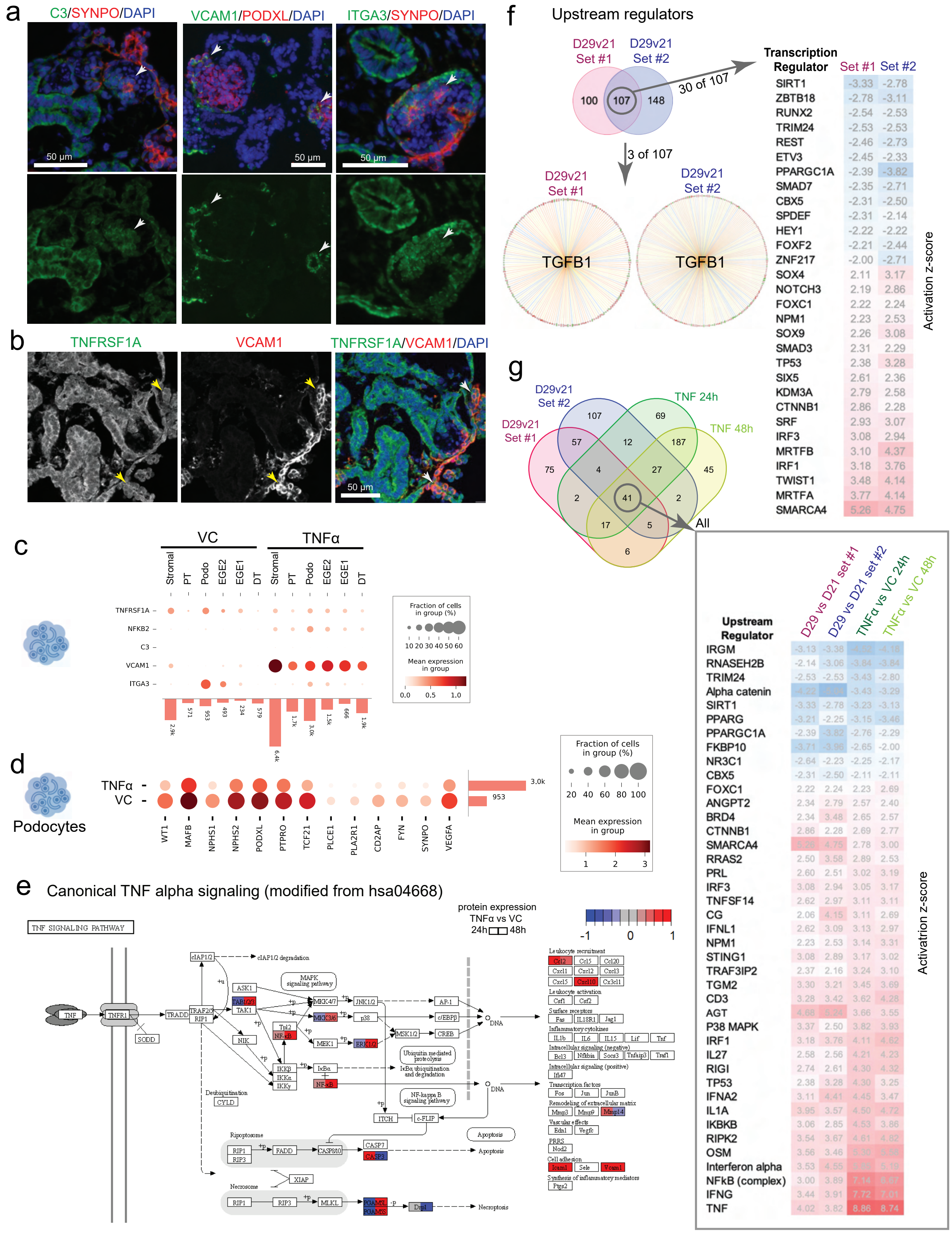

Supplemental Figure 4 | Kidney organoids express TNF receptors and known TNF downstream targets. A) Immunofluorescence imaging of kidney organoids treated with TNFα showing expression of complement factor C3 (left column), vascular cell adhesion molecule 1 (VCAM1, middle column) and Integrin Subunit Alpha 3 (ITGA3, right column) in podocytes (white arrows) expressing synaptopodin (SYNPO) and podocalyxin (PODXL) n=3, representative images shown. B) Immunofluorescence imaging of kidney organoids treated with TNFα showing expression of VCAM1 in TNFRSF1A-expressing cells (yellow arrows). DAPI, nuclear stain; n=3, representative images shown, scale bar: 50 µm. C) Dot plots showing transcript expression of (C) indicated genes in kidney cell types, and (D) podocyte markers in organoid podocytes in VC and TNFα-treated kidney organoids (Fig. 2C). E) Integration of proteins measured in TNF α-treated organoids with Kyoto Encyclopedia of Genes and Genomes (KEGG) pathway graphs. The log2 fold-changes at 24h (left side of each box) and 48h (right side of each box) were mapped onto TNF signaling pathway (KEGG-ID: hsa04668). F) Venn diagram showing overlap of 107 upstream regulators in maturing organoid datasets; (bottom) mechanistic networks showing direct connection of TGFB1 to 174 and 228 genes in each set respectively; and (right) heatmap of 30 transcription regulators within this overlap. G) Venn diagram and heatmap of 41 shared upstream regulators from (F) and TNFα-treated organoids. Source data are provided as a Source Data file.

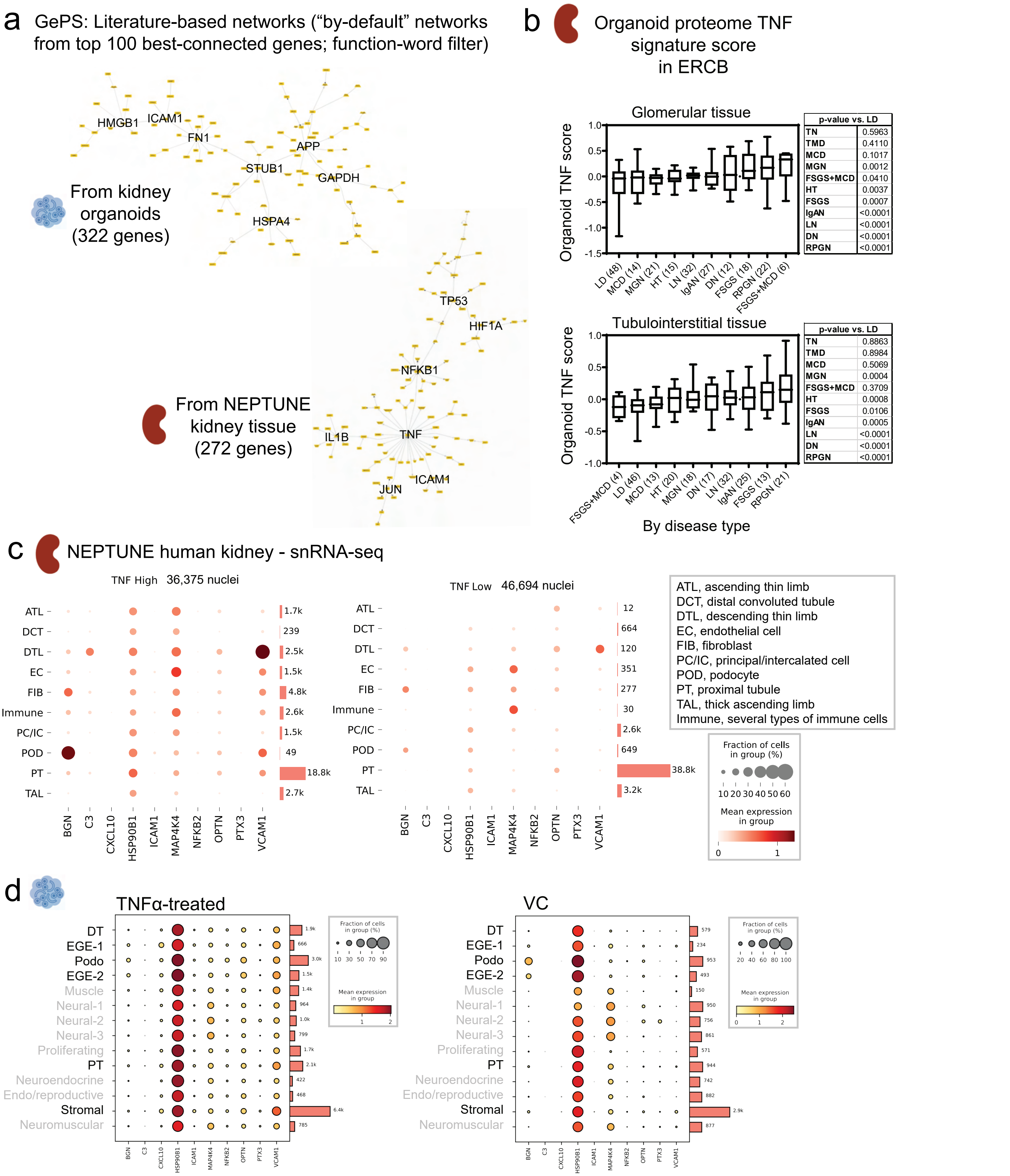

Supplemental Figure 5 | Proteins expressed by TNFα-treated kidney organoids reveal downstream activity relevant to human kidney diseases.

A) Comparison of top gene networks identified separately in the two TNF signatures, 322 gene organoid protein-based signature (left) and the 272 gene kidney tissue-based signature (right). B) Z-scores showing summary expression of organoid TNF signature genes in human kidney diseases, generated from ERCB microarray data from microdissected human kidney biopsy tissue (for the 268 of the 322 genes assessed). Glomeruli (top) and tubulointerstitia (bottom). Plots: median, boxes 25-75% percentile, whiskers represent the min. to max. values. Unpaired t-test. Numbers of individual samples per disease type are indicated in parentheses. C) Dot plots showing differential expression of the ten overlap genes of TNF signatures (as in Fig. 5C) in C) individuals with FSGS/MCD from snRNA-seq data sorted by TNFα activity (as in Figs. 5G/H) and in D) TNFα- and VC-treated kidney organoids from scRNA-seq data (as in Fig. 2C); off-target cell type cluster names are in gray. Source data are provided as a Source Data file.

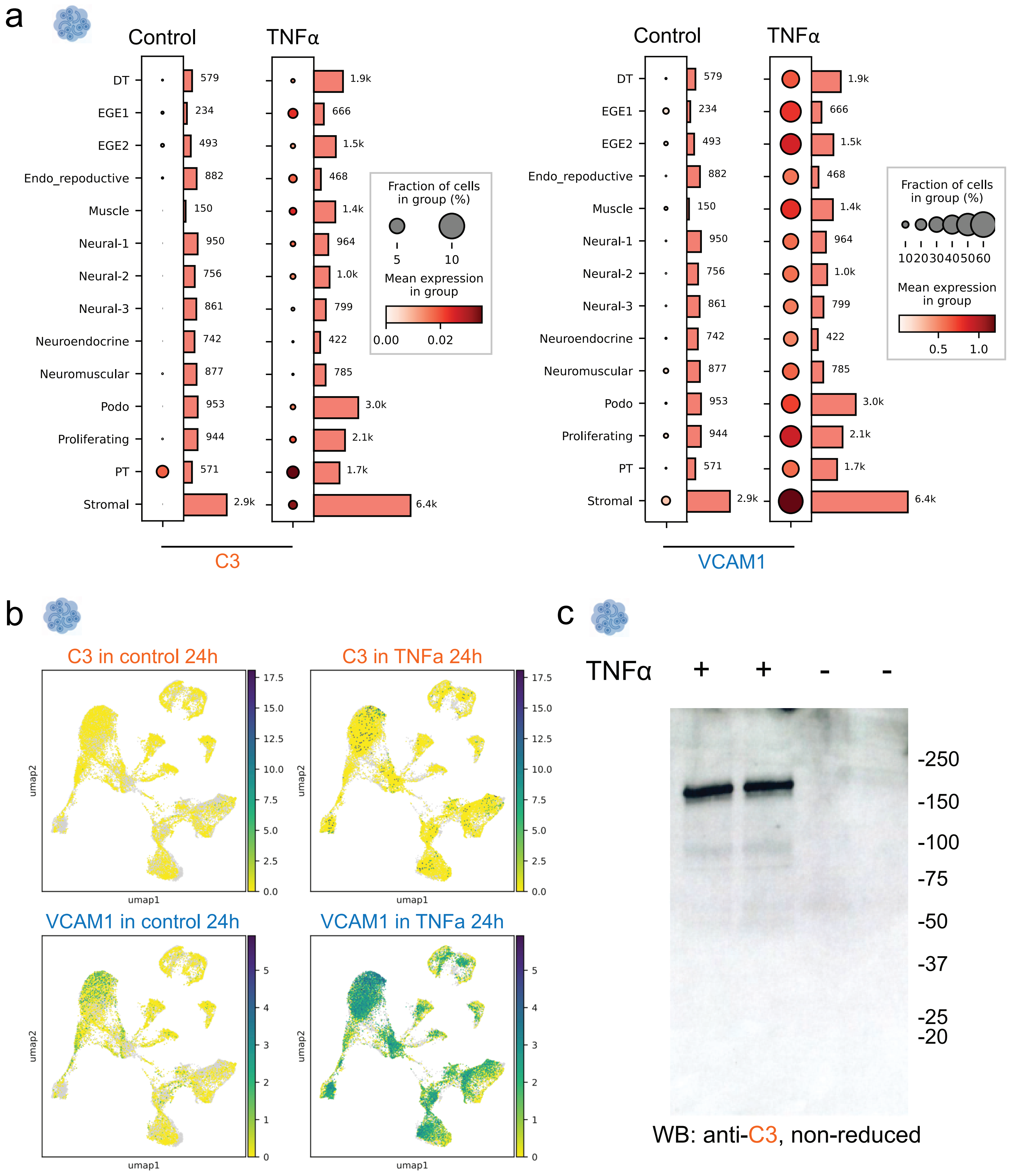

Supplemental Figure 6 | Organoid cell types express C3 and VCAM1.
A) Dot plots and B) UMAPs of scRNA-seq data from 24h VC- and TNFα-treated kidney organoids showing increased C3 and VCAM1 transcript expression in all organoid cell types (Figs 2C, 4A). C) Western blot showing expression of complement factor C3 was observed as full-length protein at 190 kDa in the supernatant of the TNFα-stimulated organoids. The western blot examining the expression of complement factor C3 was performed two times with similar result.

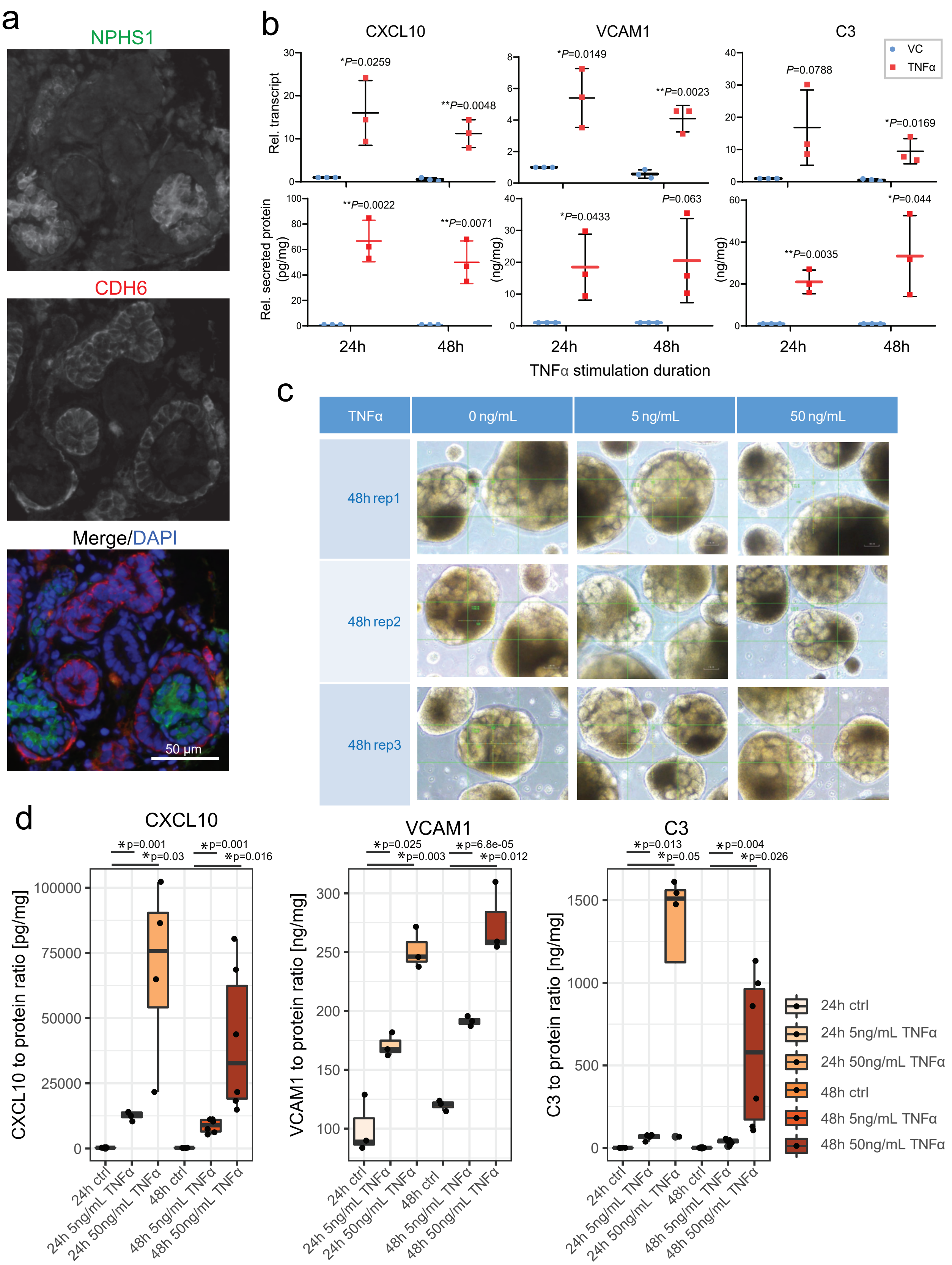

Supplemental Figure 7 | Different human iPSC lines and different protocols for organoid differentiation yield similar structures and express CXCL10, C3 and VCAM1 upon TNFα stimulation.

A) Immunofluorescence imaging of sectioned kidney organoids generated from NEPTUNE 19A cell line showing expression of nephrin (NPHS1) and k-Cadherin (CDH6), and nuclear marker DAPI; n=3, representative images shown, scale bar: 50 µm. B) Expression of CXCL10, VCAM1 and C3 increased in D24 kidney organoids (A) treated with TNFα for 24h and 48h, as measured in cell lysates by qRT-PCR (top) and in organoid culture supernatants by ELISA (bottom). Means of 3 separate experiments indicated by bold horizontal lines, with SEM error bars. Unpaired t-test. C) Light microscopy images of kidney organoids generated in suspension from UKEi001-A cell line, treated with TNFα at 5 or 50 ng/mL for 48h; treatment days 23 to 25 of organoid culture, scale bar: 100µm. D) Secretion of CXCL10, VCAM1 and C3 in organoids (C) treated with VC or TNFα at 5 or 50 ng/mL for 24 or 48h, as measured in organoid culture supernatants by ELISA (n=4-6). Plots: median, boxes represent the interquartile range (IQR, 25-75% percentile), whiskers represent the largest/smallest values within 1.5 x IQR. Treatment comparison against the respective control was carried out using t-test, adjusted p-values are shown. Source data are provided as a Source Data file.

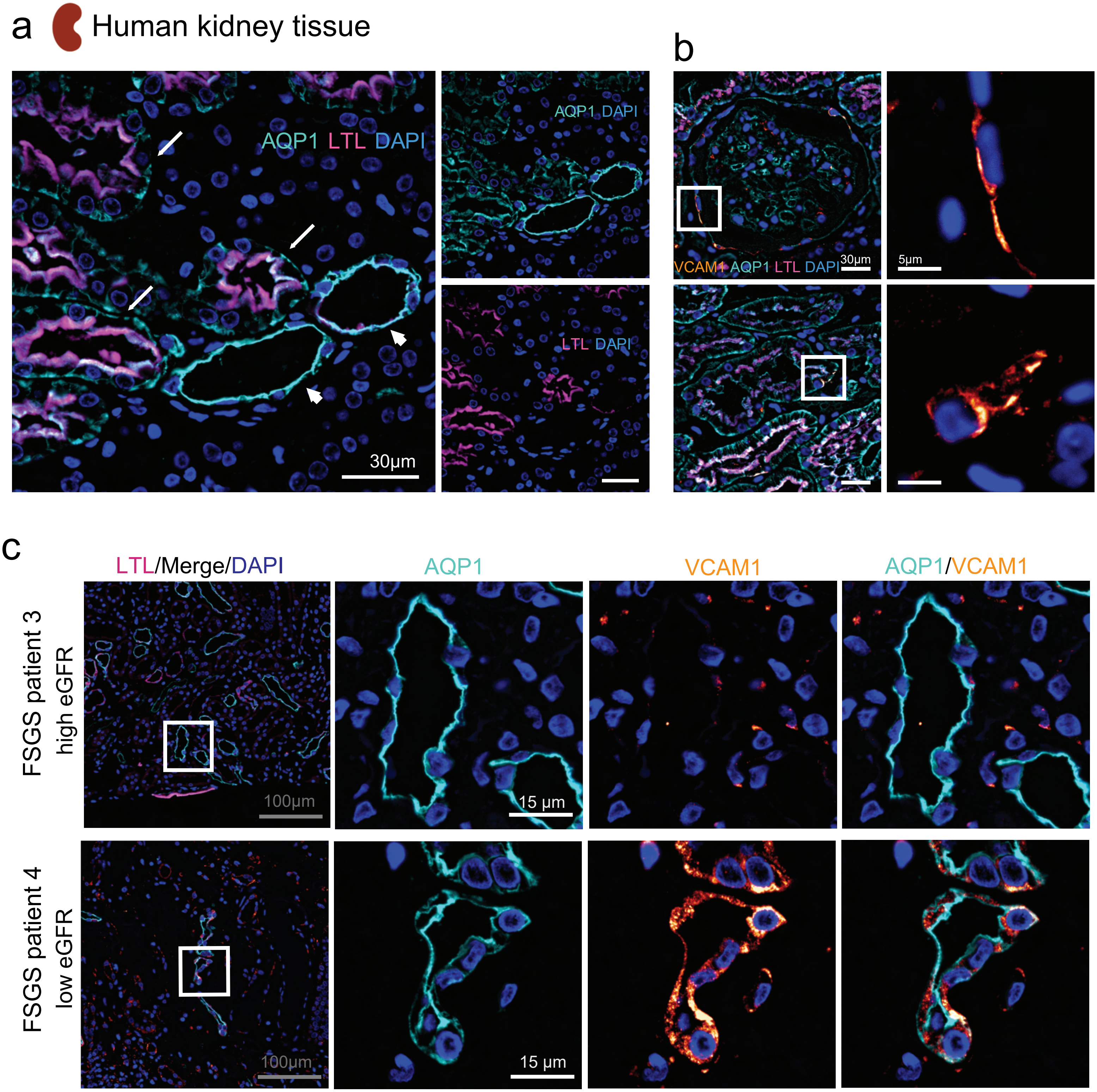

Supplemental Figure 8 | Immunofluorescence imaging of sectioned human kidney biopsies of individuals with FSGS

A) Tissue-based identification of the descending thin limb (DTL) compartment in human kidney sections. Proximal Tubules were defined as AQP1+/LTL+ tubules (thin long arrows), whereas DTL were defined as AQP1+/LTL- tubules (short arrowheads). B) Baseline expression of VCAM1 in renal compartments. Low-grade expression of VCAM1 is detectable in single glomerular parietal epithelial cells within the glomerular compartment (top row) as well as in isolated proximal tubular cells (bottom row). C) Immunofluorescence imaging of sectioned human kidney biopsies from individuals with FSGS showing expression of VCAM1 in the descending thin limb (DTL) compartment in patient 4 (eGFR = 17 mL/min/1.73m2) compared to patient 3 (eGFR = 115 mL/min/1.73m2) where no VCAM1 expression was observed. DAPI, nuclear stain. Staining conditions were optimized in human nephrectomy tissue. 10 patient samples were stained once due to limited human biobank samples. Representative images are shown. Source data are provided as a Source Data file.

a

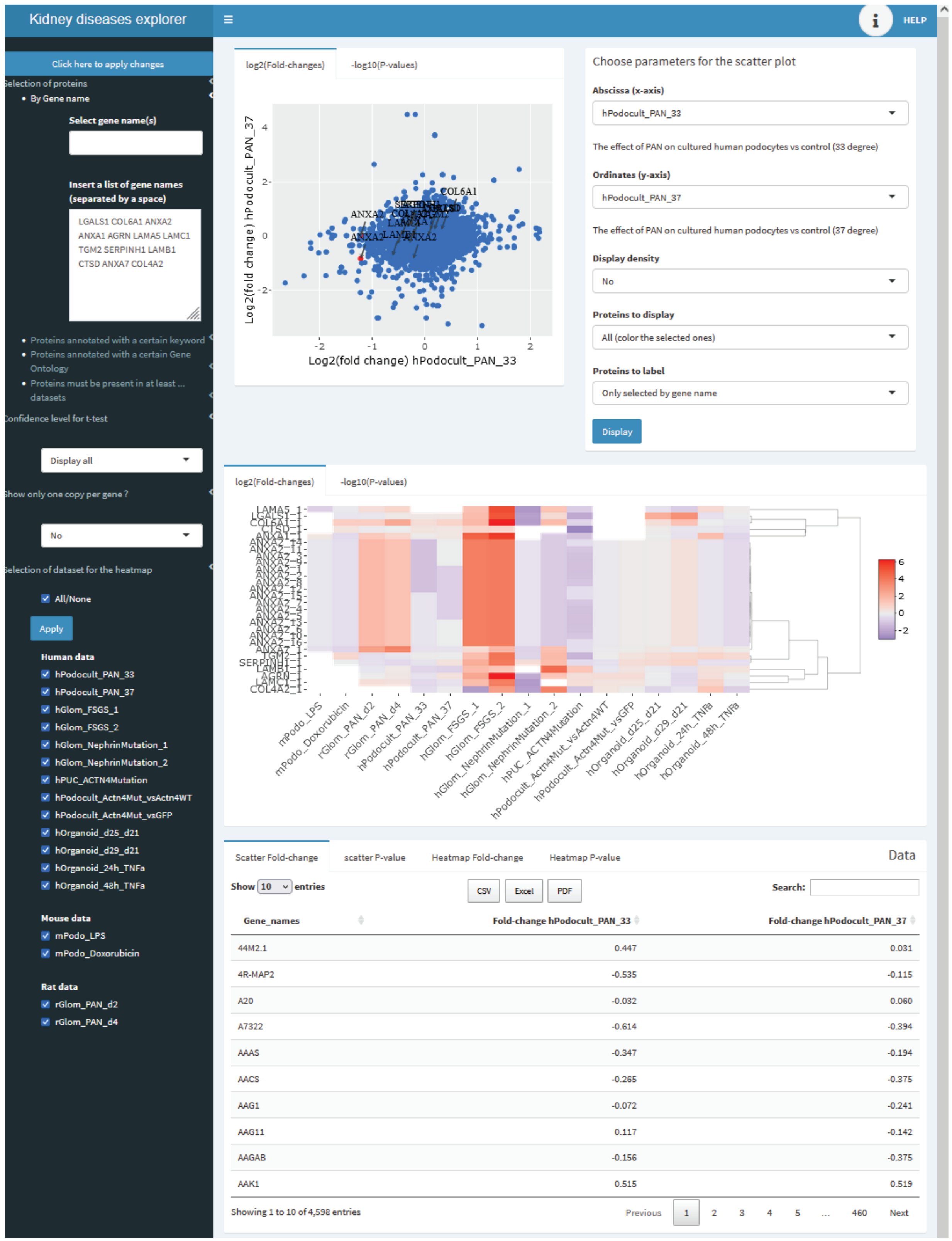

Supplemental Figure 9 | Screenshot of Kidney Disease Explorer

A) We created Kidney Disease Explorer to gain insights in the organoid data acquired here and compare it with other datasets of glomerular diseases https://kidneyapp.shinyapps.io/kidneyorganoids/. The application has four main functionalities: i) Comparing proteins from 2 datasets: display a scatterplot containing one point for each protein that is in both datasets. One can choose to display log2 of t-test's differences (log2(Fold-changes)), or -log2 of t-test's p-values (-log2(p-values)). Plot can be downloaded as PNG and SVG. ii) Comparing statistics for a selected set of proteins and datasets: display a heatmap with datasets on the y-axis and gene names on the y-axis. One can choose to display log2 of t-test's differences (log2(Fold-changes)), or -log2 of t-test's p-values (-log2(p-values)). Plot can be downloaded as PNG and SVG. iii) Visualizing selected data in a data table and downloading them as CSV, Microsoft Excel, or PDF. iv) Downloading the original dataset as CSV: for each dataset there are three variables: Gene name, ttest_difference1 which is log2 of t-test's fold changes and ttest_pvalue1 which is -log2 of t-test's p-value.

Members of the Nephrotic Syndrome Study Network (NEPTUNE)

NEPTUNE Sites
Atrium Health Levine Children's Hospital, Charlotte, SC: Susan Massengill*, Layla Lo#
Cleveland Clinic, Cleveland, OH: Katherine Dell*, John Sedor**, Stephanie Larson#
Children's Hospital, Los Angeles, CA: Ian Macumber* Kevin Lemley*, Silpa Sharma#
Children's Mercy Hospital, Kansas City, MO: Tarak Srivastava*, Kelsey Markus#
Cohen Children's Hospital, New Hyde Park, NY: Christine Sethna*, Suzanne Vento #
Columbia University, New York, NY: Pietro Canetta*, Anup Pradhan#
Duke University Medical Center, Durham, NC: Opeyemi Olabisi*, Rasheed Gbadegesin**, Maurice Smith#
Emory University, Atlanta, GA: Laurence Greenbaum*, Chia-shi Wang*, Emily Yun#
The Lundquist Institute, Torrance, CA: Sharon Adler*, Janine LaPage#
John H Stroger Cook County Hospital, Chicago, IL: Amatur Amarah*, Matthew Itteera#
Johns Hopkins Medicine, Baltimore, MD: Meredith Atkinson*, Miahje Williams#
Mayo Clinic, Rochester, MN: John Lieske*, Marie Hogan**
Medical University of South Carolina, David Selewski*, Cheryl Alston#
Montefiore Medical Center, Bronx, NY: Frederick Kaskel*, Kim Reidy**, Michael Ross*, Patricia Flynn#
NIDDK Intramural, Bethesda MD: Jeffrey Kopp**
New York University Medical Center, New York, NY: Laura Malaga-Dieguez*, Olga Zhdanova**, Laura Jane Pehrson#, Melanie Miranda#
The Ohio State University College of Medicine, Columbus, OH: Salem Almaani*, Laci Roberts#
Stanford University, Stanford, CA: Richard Lafayette*, Shiktij Dave#
Temple University, Philadelphia, PA: Iris Lee**
Texas Children's Hospital at Baylor College of Medicine, Houston, TX: Shweta Shah*, Aisha Deslandes# #
University Health Network Toronto: Heather Reich*, Michelle Hladunewich**, Paul Ling#, Martin Romano#
University of California at San Francisco, San Francisco, CA: Paul Brakeman*
University of Colorado Anschutz Medical Campus, Aurora, CO: Amber Podoll* Nathan Rogers#
University of Kansas Medical Center, Kansas City, KS: Ellen McCarthy*, Elizabeth Landry#
University of Miami, Miami, FL: Alessia Fornoni*, Carlos Bidot#
University of Michigan, Ann Arbor, MI: M Kretzler*, Laura Mariani*, Zubin Modi*, A Williams#, Meghan Stelzer#
University of Minnesota, Minneapolis, MN: Patrick Nachman*, Michelle Rheault*, Jenna Hanson#
University of North Carolina, Chapel Hill, NC: Vimal Derebail*, Keisha Gibson*, Anne Froment#
University of Pennsylvania, Philadelphia, PA: Lawrence Holzman*, Kevin Meyers**, Krishna Kallem#, Ann Swenson#
University of Texas San Antonio, San Antonio, TX: Samin Sharma**
University of Texas Southwestern, Dallas, TX: Elizabeth Roehm*, Kamalanathan Sambandam*, Jamie Hellewege
University of Washington, Seattle, WA: Ashley Jefferson*, Sangeeta Hingorani**, Katherine Tuttle**§, Linda Manahan #, Emily Pao#, Kelli Kuykendall§
Wake Forest University Baptist Health, Winston-Salem, NC: Jen Jar Lin**
Washington University in St. Louis, St. Louis, MO: Vikas Dharnidharka*

Data Analysis and Coordinating Center: Matthias Kretzler*, Laura Barisoni**, Crystal Gadegbeku**, Brenda Gillespie**, Lawrence Holzman**, Laura Mariani**, Zubin Modi**, Matthew G Sampson**, Eloise Salmon**, John Sedor**, Abigail Smith**, Howard Trachtman**, Jarcy Zee**, Gabrielle Alter, Hailey Desmond, Sean Eddy, Damian Fermin, Wenjun Ju, Maria Larkina, Shengqian Li, Shannon Li, Chrysta Lienczewski, Tina Mainieri, Rebecca Scherr, Jonathan Troost, Amanda Williams

Digital Pathology Committee: Carmen Avila-Casado (University Health Network, Toronto), Serena Bagnasco (Johns Hopkins University), Clarissa Cassol (Arakana), Lihong Bu (Mayo Clinic), Shelley Caltharp (Emory University), Dawit Demeke (University of Michigan), Brenda Gillespie (University of Michigan), Jared Hassler (Temple University), Leal Herlitz (Cleveland Clinic), Stephen Hewitt (National Cancer Institute), Jeff Hodgin (University of Michigan), Danni Holanda (Arkana), Neeraja Kambham (Stanford University), Kevin Lemley (Children's Hospital of Los Angeles), Laura Mariani (University of Michigan), Nidia Messias

*Principal Investigator; **Co-investigator; #Study Coordinator; §Providence Medical Research Center, Spokane, WA

Last Update: 25 May 2023

(Washington University), Alexei Mikhailov (Wake Forest), Behzad Najafian (University of Washington), Matthew Palmer (University of Pennsylvania), Avi Rosenberg (Johns Hopkins University), Virginie Royal (University of Montreal), Barry Stokes (Columbia University), David Thomas (Duke University), Michifumi Yamashita (Cedar Sinai), Hong Yin (Emory University) Jarcy Zee (University of Pennsylvania), Yiqin Zuo (University of Miami) Co-Chairs: Laura Barisoni (Duke University) and Cynthia Nast (Cedar Sinai).

ERCB Members at the time of the study:

Clemens David Cohen, Holger Schmid, Michael Fischereder, Lutz Weber, Matthias Kretzler, Detlef Schlöndorff, Munich/Zurich/AnnArbor/NewYork;
Jean Daniel. Sraer, Pierre Ronco, Paris;
Maria Pia Rastaldi, Giuseppe D'Amico, Milano;
Peter Doran, Hugh Brady, Dublin;
Detlev Mönks, Christoph Wanner, Würzburg;
Andrew Rees, Aberdeen and Vienna;
Frank Strutz, Gerhard Anton Müller, Göttingen;
Peter Mertens, Jürgen Floege, Aachen;
Norbert Braun, Teut Risler, Tübingen;
Loreto Gesualdo, Francesco Paolo Schena, Bari;
Gunter Wolf, Jena;
Rainer Oberbauer, Dontscho Kerjaschki, Vienna;
Bernhard Banas, Bernhard Krämer, Regensburg;
Moin Saleem, Bristol;
Rudolf Wüthrich, Zurich;
Walter Samtleben, Munich;
Harm Peters, Hans-Hellmut Neumayer, Berlin;
Mohamed Daha, Leiden;
Katrin Ivens, Bernd Grabensee, Düsseldorf;
Francisco Mampaso(†), Madrid;
Jun Oh, Franz Schaefer, Martin Zeier, Hermann-Joseph Gröne, Heidelberg;
Peter Gross, Dresden;
Giancarlo Tonolo; Sassari;
Vladimir Tesar, Prague;
Harald Rupprecht, Bayreuth;
Hermann Pavenstädt,
Münster; Hans-Peter Marti, Bern;
Peter Mertens, Magdeburg,
Jens Gerth, Zwickau.
