# Supplementary material for: An integrated organoid omics map extends modeling potential of kidney disease
Source: Nat Commun. 2023 Aug 14;14:4903. doi: 10.1038/s41467-023-39740-7 (PMC10425428; doi:10.1038/s41467-023-39740-7)
Supplement: Supplementary file 3 — Description of Additional Supplementary Files [file 41467_2023_39740_MOESM3_ESM.pdf]

Description of Additional Supplementary Files

Supplementary Data 1. organoids proteome, >6700 proteins observed

Supplementary Data 2. organoid proteins used for time course analysis quantification

Supplementary Data 3. organoid RNA used for time course analysis quantification (set #1)

Supplementary Data 4. organoid cell types as clustered by scRNAseq data UMAP

Supplementary Data 5. organoid proteins from the cell pellet, after stimulation with 5ng/mL TNFa for 24h and 48h

Supplementary Data 6. organoids media-only proteins

Supplementary Data 7. organoid proteins from the supernatant, after stimulation with 5ng/mL TNFa for 24h and 48h

Supplementary Data 8. Moins cultured podocyte proteins from the cell pellet, after stimulation with 5ng/mL TNFa for 24h and 48h

Supplementary Data 9. Moins cultured podocyte proteins from the supernatant, after stimulation with 5ng/mL TNFa for 24h and 48h

Supplementary Data 10. organoid derived TNFa signature

Supplementary Data 11. top 20 GePS pathway based networks $p < 0.05$

Supplementary Data 12. top 20 GePS signal transduction networks $p < 0.05$

Supplementary Data 13. receptors in organoids on protein level

Supplementary Data 14. organoid RNA used for time course analysis quantification (set #2)

Supplementary Data 15. organoid RNA, after stimulation with 5 ng/mL TNFa for 24h and 48h

Supplementary Data 16. upstream regulators day 29 versus day 21 organoids from IPA

Supplementary Data 17. downstream mocules from TGFB1 mechanistic networks day 29 versus day 21 organoids from IPA

Supplementary Data 18. upstream regulators organoids treated with TNF or VC for 24h and 48h from IPA

Supplementary Data 19. Organoid TNF signature genes interrogated in NEPTUNE kidney tissue transcriptome
