# Supplementary material for: An integrated organoid omics map extends modeling potential of kidney disease
Source: Nat Commun. 2023 Aug 14;14:4903. doi: 10.1038/s41467-023-39740-7 (PMC10425428; doi:10.1038/s41467-023-39740-7)
Supplement: Supplementary file 5 — Reporting Summary [file 41467_2023_39740_MOESM5_ESM.pdf]

nature portfolio

Corresponding author(s): Jennifer L. Harder

Last updated by author(s): May 22, 2023

Reporting Summary

Nature Portfolio wishes to improve the reproducibility of the work that we publish. This form provides structure for consistency and transparency in reporting. For further information on Nature Portfolio policies, see our Editorial Policies and the Editorial Policy Checklist.

Statistics

For all statistical analyses, confirm that the following items are present in the figure legend, table legend, main text, or Methods section.

n/a	Confirmed	
☐	☒	The exact sample size (*n*) for each experimental group/condition, given as a discrete number and unit of measurement
☐	☒	A statement on whether measurements were taken from distinct samples or whether the same sample was measured repeatedly
☐	☒	The statistical test(s) used AND whether they are one- or two-sided<br>*Only common tests should be described solely by name; describe more complex techniques in the Methods section.*
☒	☐	A description of all covariates tested
☐	☒	A description of any assumptions or corrections, such as tests of normality and adjustment for multiple comparisons
☐	☒	A full description of the statistical parameters including central tendency (e.g. means) or other basic estimates (e.g. regression coefficient) AND variation (e.g. standard deviation) or associated estimates of uncertainty (e.g. confidence intervals)
☐	☒	For null hypothesis testing, the test statistic (e.g. *F*, *t*, *r*) with confidence intervals, effect sizes, degrees of freedom and *P* value noted<br>*Give P values as exact values whenever suitable.*
☒	☐	For Bayesian analysis, information on the choice of priors and Markov chain Monte Carlo settings
☒	☐	For hierarchical and complex designs, identification of the appropriate level for tests and full reporting of outcomes
☒	☐	Estimates of effect sizes (e.g. Cohen's *d*, Pearson's *r*), indicating how they were calculated

Our web collection on statistics for biologists contains articles on many of the points above.

Software and code

Policy information about availability of computer code

Data collection	All software and code has been previously published and is described in the methods section of the paper.
Data analysis	All software and code has been previously published and is described in the methods section of the paper.

For manuscripts utilizing custom algorithms or software that are central to the research but not yet described in published literature, software must be made available to editors and reviewers. We strongly encourage code deposition in a community repository (e.g. GitHub). See the Nature Portfolio guidelines for submitting code & software for further information.

Data

Policy information about availability of data

All manuscripts must include a data availability statement. This statement should provide the following information, where applicable:

- Accession codes, unique identifiers, or web links for publicly available datasets
- A description of any restrictions on data availability
- For clinical datasets or third party data, please ensure that the statement adheres to our policy

Data Availability
Organoid bulk and single cell RNA-seqdata were uploaded to NCBI GEO accession number GSE213972 . A Cell x Gene visualization instance is available here:http://18.188.163.197/(username: reviewer; password: POhA1neVjLKem)Proteomics analysis, organoid time course. The mass spectrometry proteomics data have been deposited to the ProteomeXchange Consortium via the PRIDE 54partner repository with the dataset identifiers. Project Name: LC MS/MS of kidney

organoids during differentiation from day 21 to 29 Project accession: PXD029716. https://www.ebi.ac.uk/pride/archive/projects/PXD029716Proteomic analysis of TNFα-treated organoids. The mass spectrometry proteomics data have been deposited to the ProteomeXchange Consortium via the PRIDE 54partner repository with the dataset identifiers. Project Name 1 (organoid spheroid cell lysate): Proteome analysis of kidney organoid cells during TNFα stimulation Project accession: PXD029718 https://www.ebi.ac.uk/pride/archive/projects/PXD029718. Project Name 2 (secretome): Proteomic analysis of kidney organoid supernatant during TNFα stimulation Project accession: PXD029696 https://www.ebi.ac.uk/pride/archive/projects/PXD029696.Analysis of the human cultured podocyte proteome. The mass spectrometry proteomics data have been deposited to the ProteomeXchange Consortium via the PRIDE 54partner repository with the dataset identifiers. Project Name 1: Proteomic analysis of cultured human podocytes stimulated with TNFα -cell pellet, Project accession: PXD032107 https://www.ebi.ac.uk/pride/archive/projects/PXD032107. Project Name 2: Proteomic analysis of cultured human podocytes stimulated with TNFα –supernatant, Project accession: PXD032130, https://www.ebi.ac.uk/pride/archive/projects/PXD032130. NEPTUNE bulk RNA-seq tubulointerstitium kidney biopsy data from NEPTUNE participants with FSGS/MCD were accessed via GEO accession number GSE182380(https://www.ncbi.nlm.nih.gov/geo/query/acc.cgi). Single nuclear RNA-seq data for ten NEPTUNE participants were accessed via GEO accession number GSE213030(https://www.ncbi.nlm.nih.gov/geo/query/acc.cgi). Transcriptomic (microarray) data of the European Renal cDNA Bank (ERCB) tissue were accessed for glomerular and tubulointerstitial compartments via NCBI GEO accession numbers GSE104948 (https://www.ncbi.nlm.nih.gov/geo/query/acc.cgi) and GSE104954(https://www.ncbi.nlm.nih.gov/geo/query/acc.cgi).No novel code was developed for this research. Source data are provided with this paper.

Research involving human participants, their data, or biological material

Policy information about studies with human participants or human data. See also policy information about sex, gender (identity/presentation), and sexual orientation and race, ethnicity and racism.

Reporting on sex and gender	Sexes of donor for 3 hPSC lines were included the manuscript. Sexes of kidney tissue biopsy sample are not reported to preserve anonymity given that diagnosis, GFR & UPC are provided.
Reporting on race, ethnicity, or other socially relevant groupings	N/A
Population characteristics	N/A
Recruitment	N/A
Ethics oversight	Details are provided in the manuscript Methods sections.

Note that full information on the approval of the study protocol must also be provided in the manuscript.

Field-specific reporting

Please select the one below that is the best fit for your research. If you are not sure, read the appropriate sections before making your selection.

☒ Life sciences ☐ Behavioural & social sciences ☐ Ecological, evolutionary & environmental sciences

For a reference copy of the document with all sections, see nature.com/documents/nr-reporting-summary-flat.pdf

Life sciences study design

All studies must disclose on these points even when the disclosure is negative.

Sample size	Where technically feasible, 3 sets of 20 organoids were evaluated for each condition queried and adjusted p-values were calculated. In some cases where samples were collected in parallel for transcriptional analysis, proteomic analysis and/or immunofluorescence imaging, sample was limited by sample availability.
Data exclusions	We excluded one TNF experiment when it was discovered that CXCL10 level did not increase as expected and it was discovered that the rTNFa used was beyond the manufacturer recommended use period; the rTNFa was replaced and the experiment repeated, with the expected results.
Replication	We repeated TNF organoid experiments 5 times with consistent results; from this group, we sent samples for bulk RNA-seq. We repeated this again 3 times using a different iPSC line, and using a 3rd iPSC line with a different organoid differentiation protocol.
Randomization	Groups of organoid wells were assigned to treatment groups in blocks to minimize technical error.
Blinding	Blinding was not relevant.

Reporting for specific materials, systems and methods

We require information from authors about some types of materials, experimental systems and methods used in many studies. Here, indicate whether each material, system or method listed is relevant to your study. If you are not sure if a list item applies to your research, read the appropriate section before selecting a response.

Materials & experimental systems

n/a	Involved in the study	
☐	☒	Antibodies
☐	☒	Eukaryotic cell lines
☒	☐	Palaeontology and archaeology
☒	☐	Animals and other organisms
☒	☐	Clinical data
☒	☐	Dual use research of concern
☒	☐	Plants

Methods

n/a	Involved in the study	
☒	☐	ChIP-seq
☒	☐	Flow cytometry
☒	☐	MRI-based neuroimaging

Antibodies

Antibodies used

Primary antibodies: N-Cadherin (R&D, cat#AF6426, 1:1000); ACTA2 (R&D clone 1A4, cat#MAB1420-SP, 1:50); PDGFRA (BD Biosciences clone αR1, cat#556001, 1:200); Synaptopodin (Progen clone G1D4, cat#690094S, 1:80); NPHS1 (R&D, cat#AF4269, 1:500); TNFARSF1A (R&D clone 16803, cat#MAB225SP, 1:20); VCAM1 (Invitrogen clone 1.4C3, cat#MA5-11447, 1:50).

0.5 µg/mL polyclonal rabbit anti-human-C3c (DAKO #0368)

Primary antibodies and their respective host species and dilutions used in this study are as follows: AQP1 (SantaCruz sc-25287, clone B-11; mouse; 1:200), VCAM1 (Abcam ab134047; clone EPR5047, rabbit; 1:200). The following secondary antibodies were used: Streptavidin Alexa Fluor 488 conjugate (Invitrogen S11223; 1:400), donkey anti-rabbit Alexa Fluor 555 conjugate (Invitrogen A31572, 1:200), donkey anti-mouse Alexa Fluor 647 conjugate (Invitrogen A31571; 1:200).

Validation

Staining patterns of organoids and kidney tissue were assessed in context of known cell type markers, and interpreted in context of single cell transcriptional expression, and reported human kidney pattern (Human Protein Atlas).

Eukaryotic cell lines

Policy information about cell lines and Sex and Gender in Research

Cell line source(s)

UM77-2 (female) hESC line was obtained from University of Michigan Stem Cell Core; NEPTUNE 19A (male) iPSC line was reprogrammed at the University of Michigan Human Stem Cell and Gene Editing Core; UKEi001A (female) iPSC line was obtained from University Medical Center Hamburg-Eppendorf.

Authentication

As described in Methods.

Mycoplasma contamination

All lines tested negative for Mycoplasma contamination.

Commonly misidentified lines
(See ICLAC register)

No commonly misidentified lines were used.
