# Supplementary figures and images for: An integrated organoid omics map extends modeling potential of kidney disease
Source: Nat Commun. 2023 Aug 14;14:4903. doi: 10.1038/s41467-023-39740-7 (PMC10425428; doi:10.1038/s41467-023-39740-7)

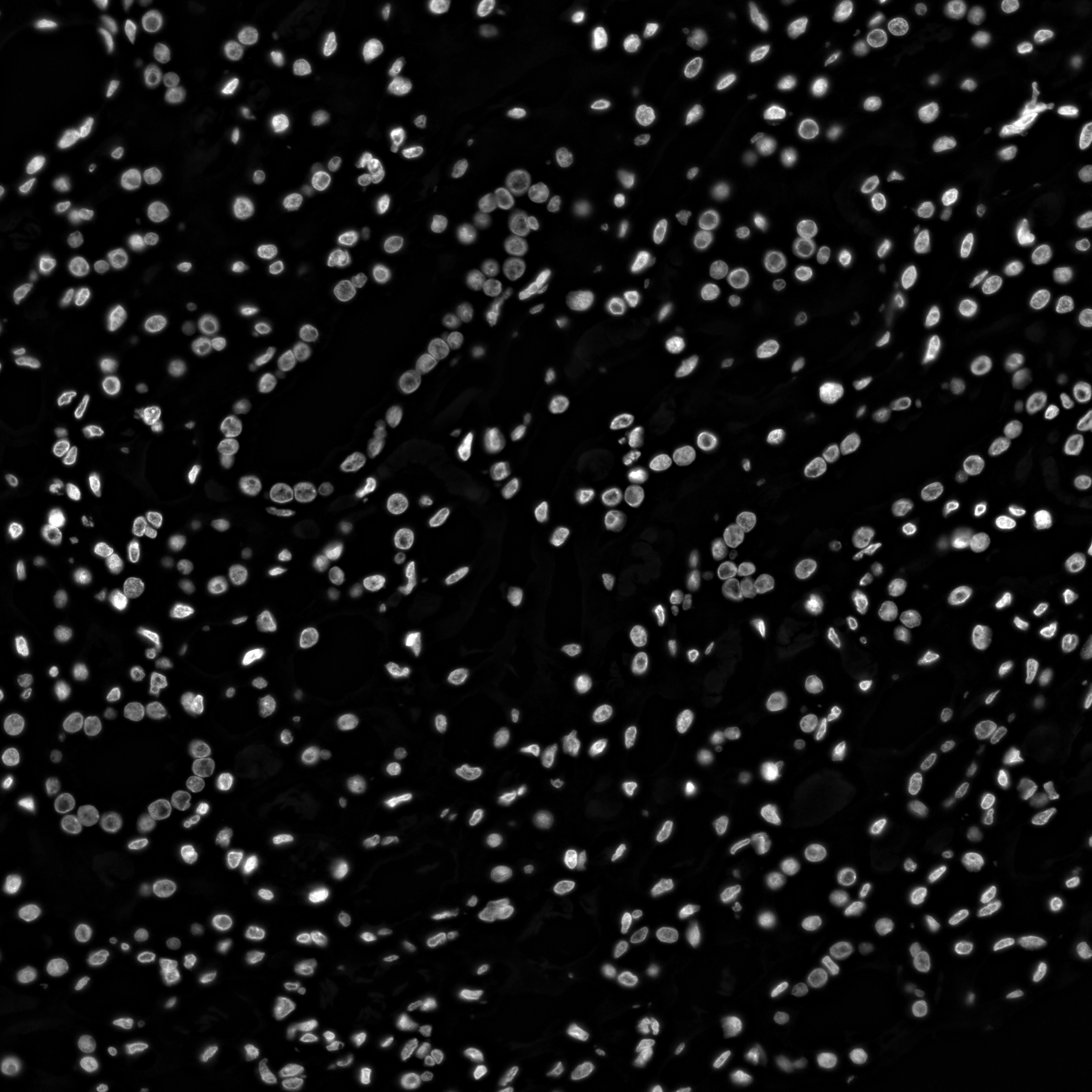

Supplement: Supplementary file 6 — Source Data [file 41467_2023_39740_MOESM6_ESM.zip › FSGS_IF/fig6f/6f pat1 overview.tif]

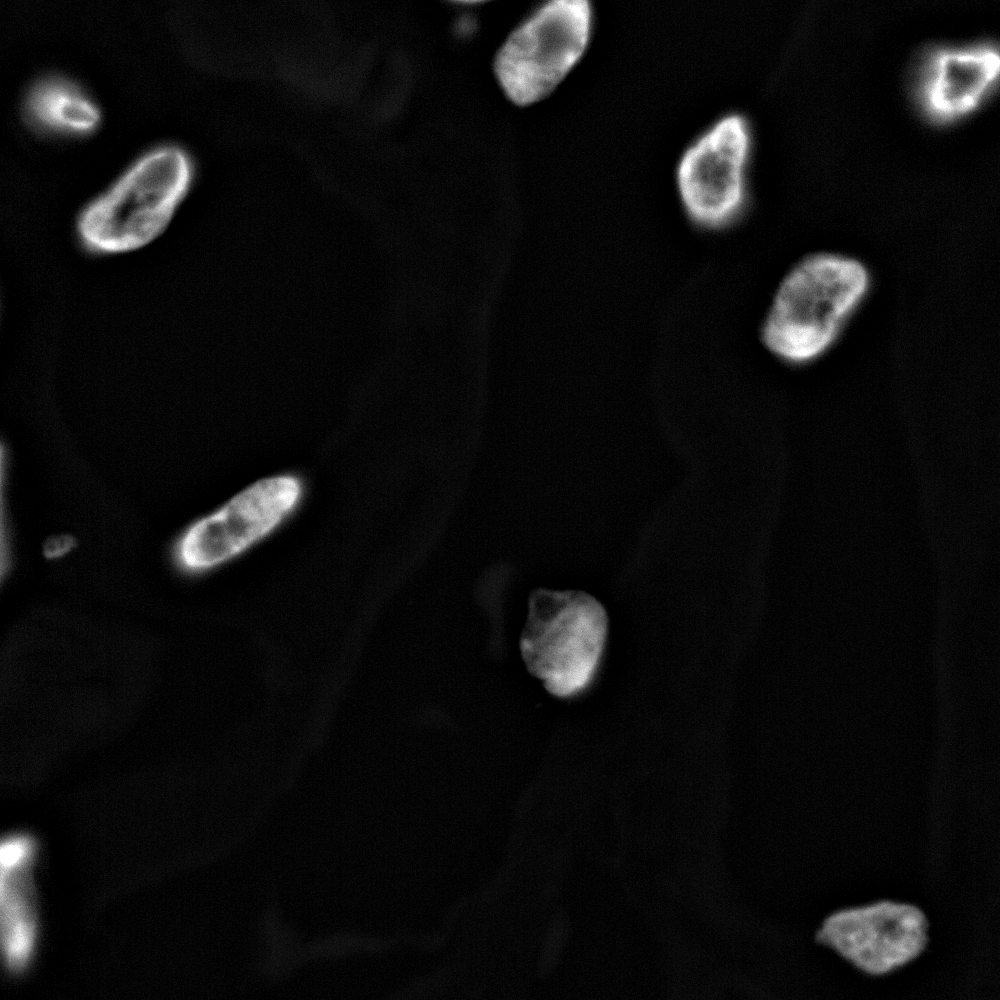

Supplement: Supplementary file 6 — Source Data [file 41467_2023_39740_MOESM6_ESM.zip › FSGS_IF/fig6f/6f pat1 region.tif]

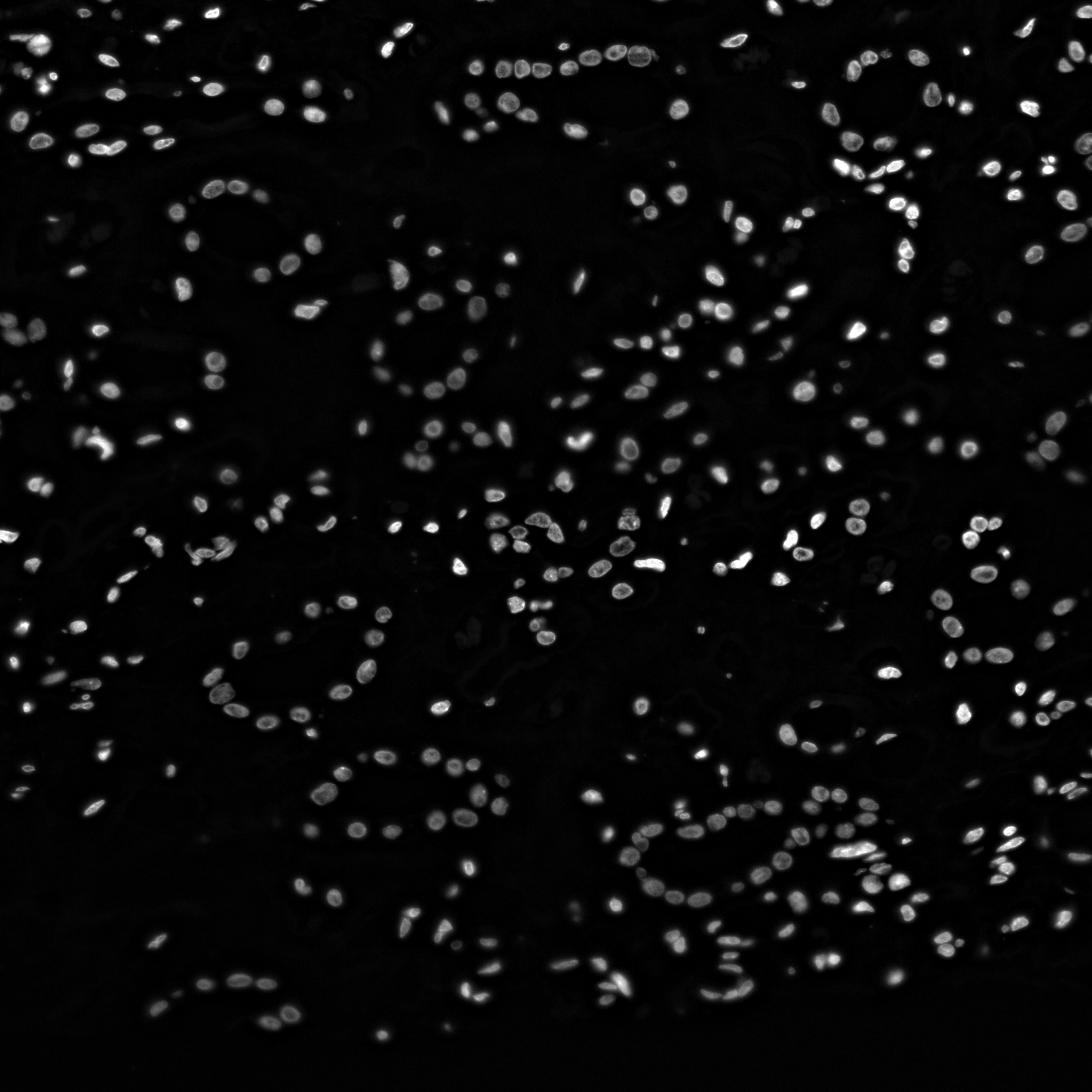

Supplement: Supplementary file 6 — Source Data [file 41467_2023_39740_MOESM6_ESM.zip › FSGS_IF/fig6f/6f pat2 overview.tif]

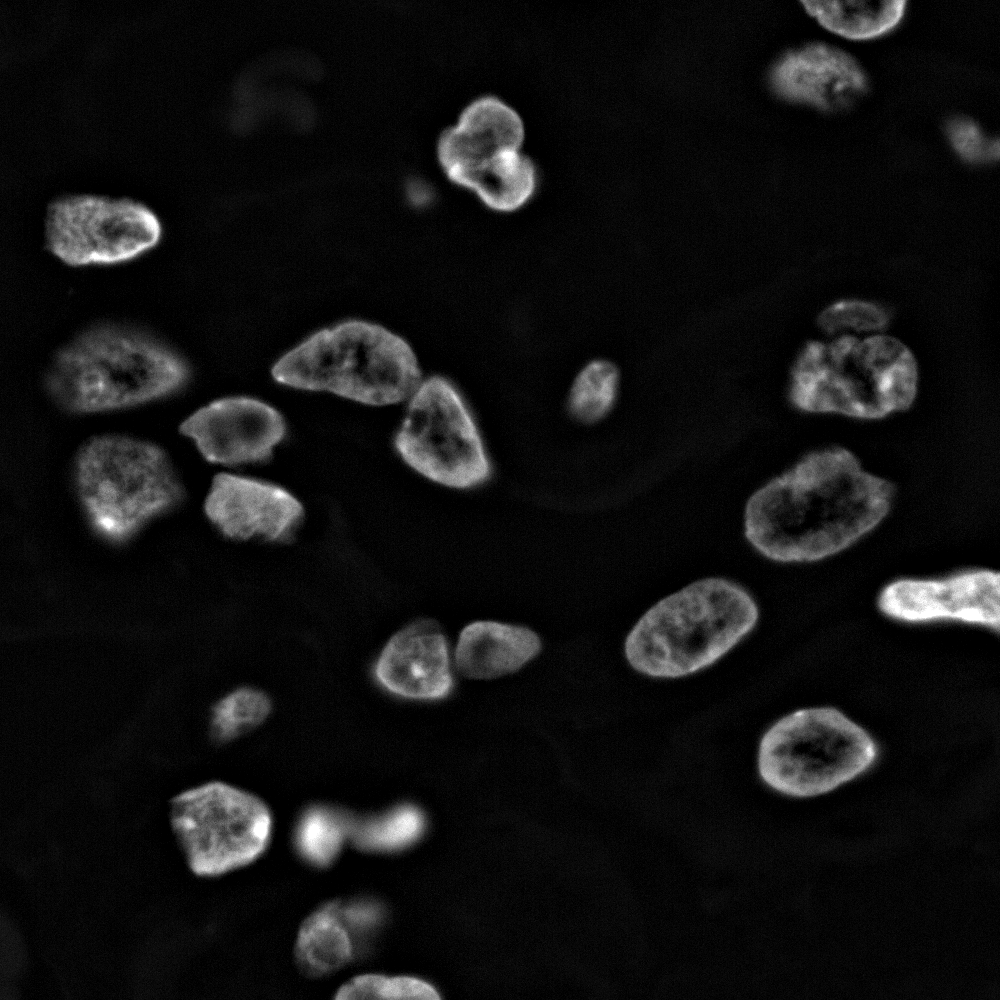

Supplement: Supplementary file 6 — Source Data [file 41467_2023_39740_MOESM6_ESM.zip › FSGS_IF/fig6f/6f pat2 region.tif]

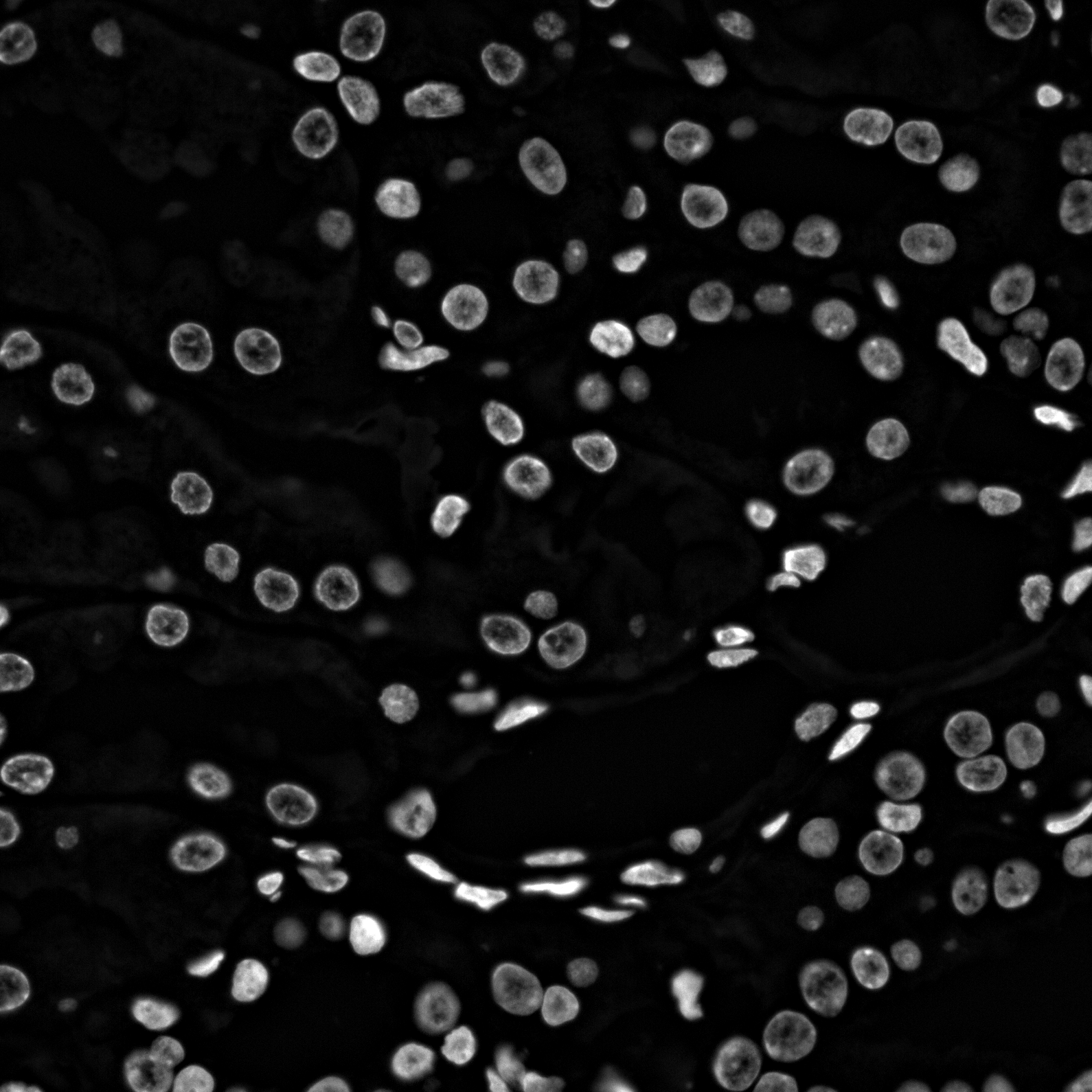

Supplement: Supplementary file 6 — Source Data [file 41467_2023_39740_MOESM6_ESM.zip › FSGS_IF/suppl_fig_8/suppl 8a.tif]

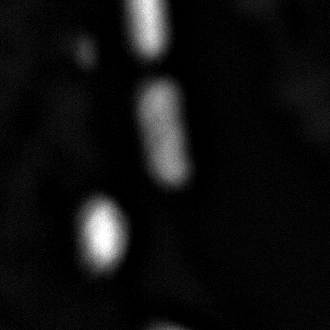

Supplement: Supplementary file 6 — Source Data [file 41467_2023_39740_MOESM6_ESM.zip › FSGS_IF/suppl_fig_8/suppl 8b glom small region.tif]

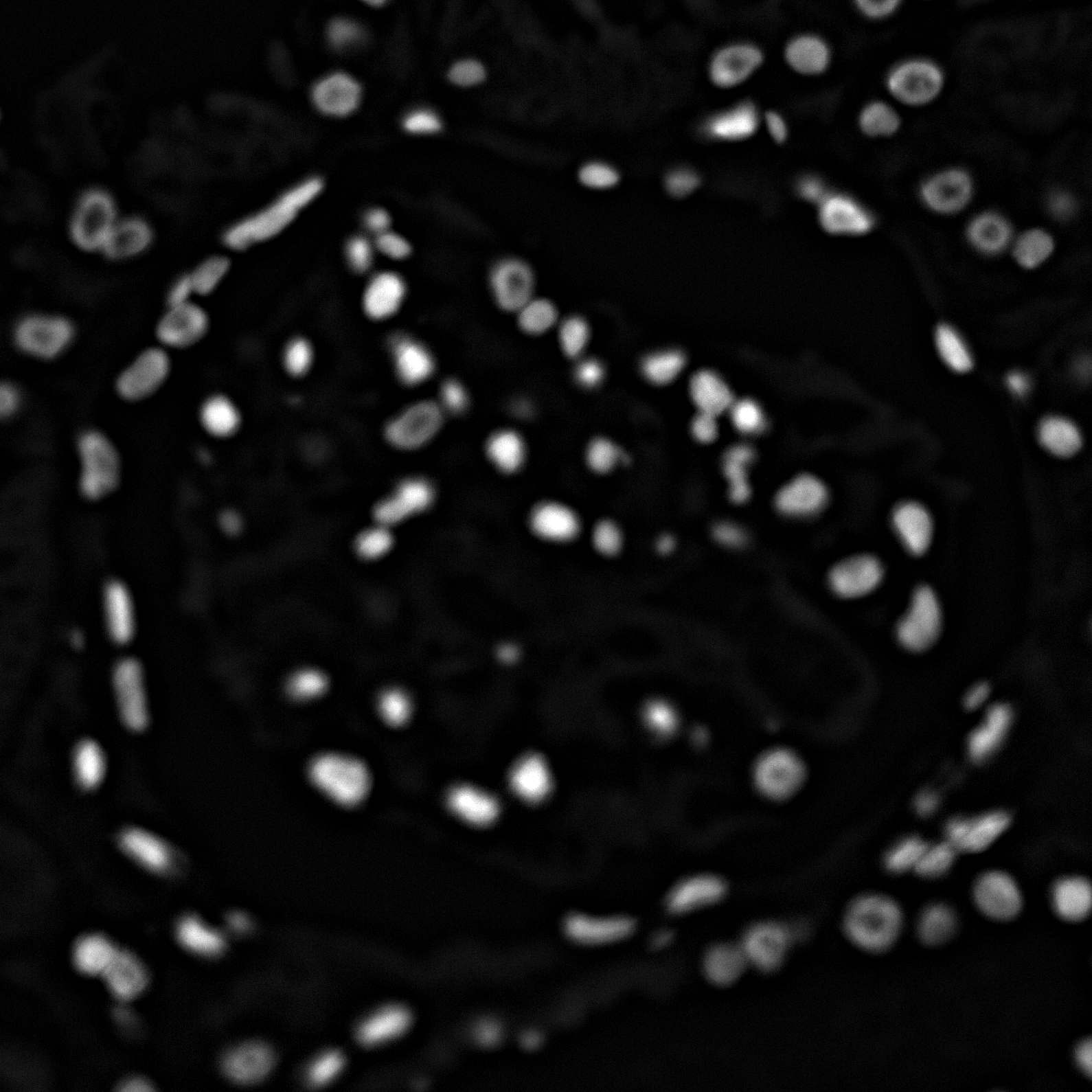

Supplement: Supplementary file 6 — Source Data [file 41467_2023_39740_MOESM6_ESM.zip › FSGS_IF/suppl_fig_8/suppl 8b glom.tif]

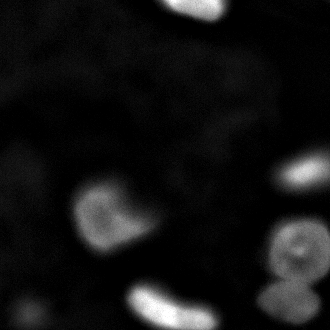

Supplement: Supplementary file 6 — Source Data [file 41467_2023_39740_MOESM6_ESM.zip › FSGS_IF/suppl_fig_8/suppl 8b tubuli small region.tif]

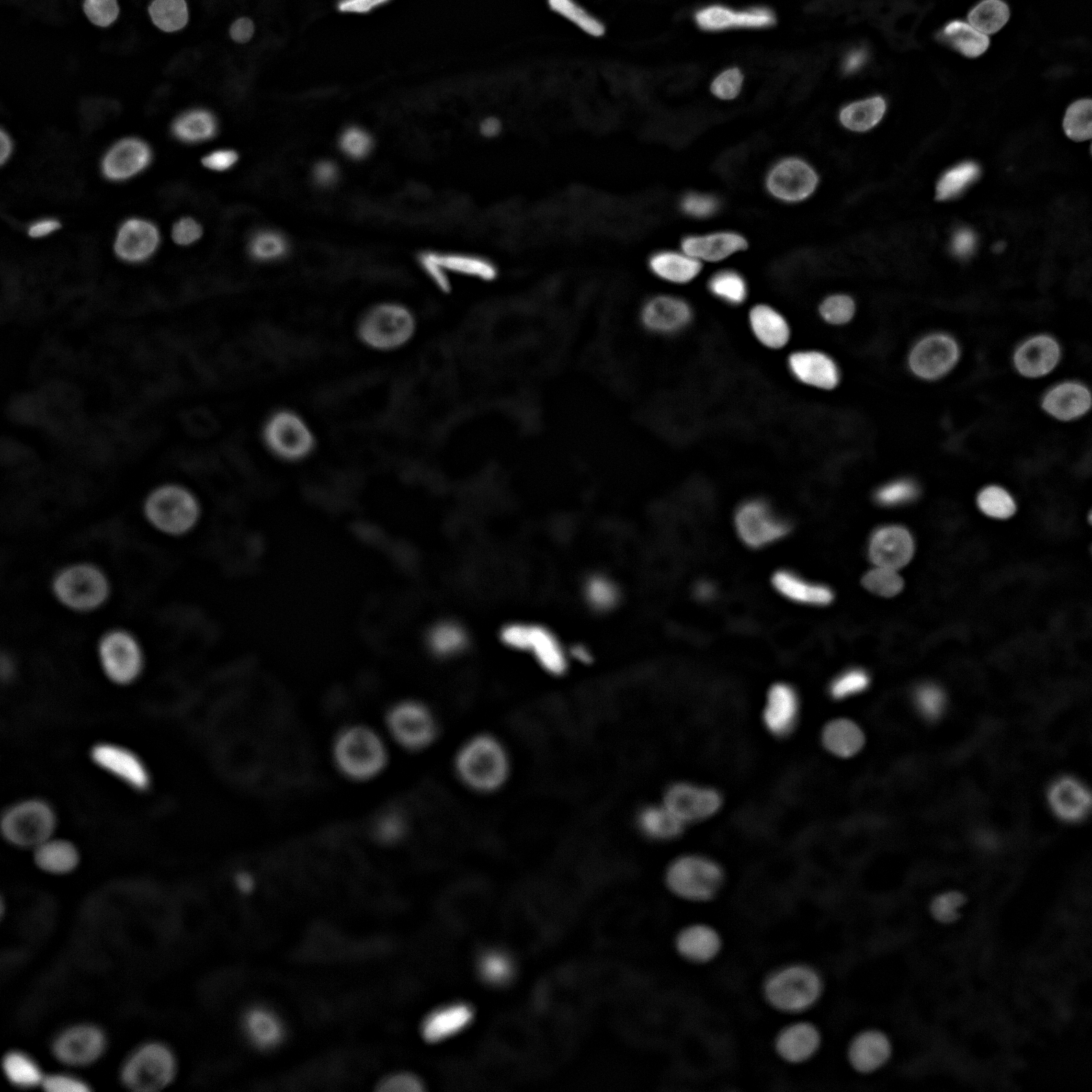

Supplement: Supplementary file 6 — Source Data [file 41467_2023_39740_MOESM6_ESM.zip › FSGS_IF/suppl_fig_8/suppl 8b tubuli.tif]

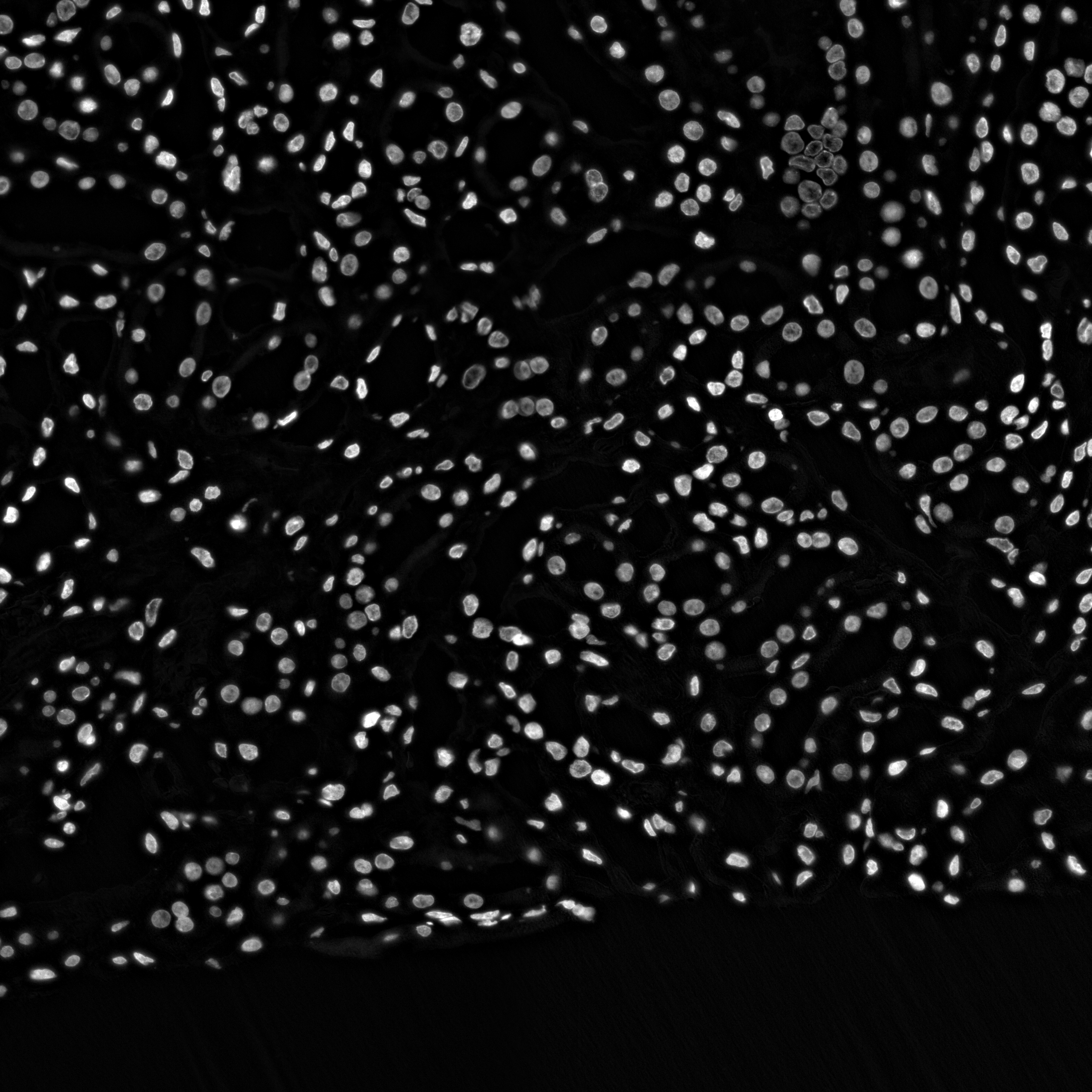

Supplement: Supplementary file 6 — Source Data [file 41467_2023_39740_MOESM6_ESM.zip › FSGS_IF/suppl_fig_8/suppl 8c pat3 overview.tif]

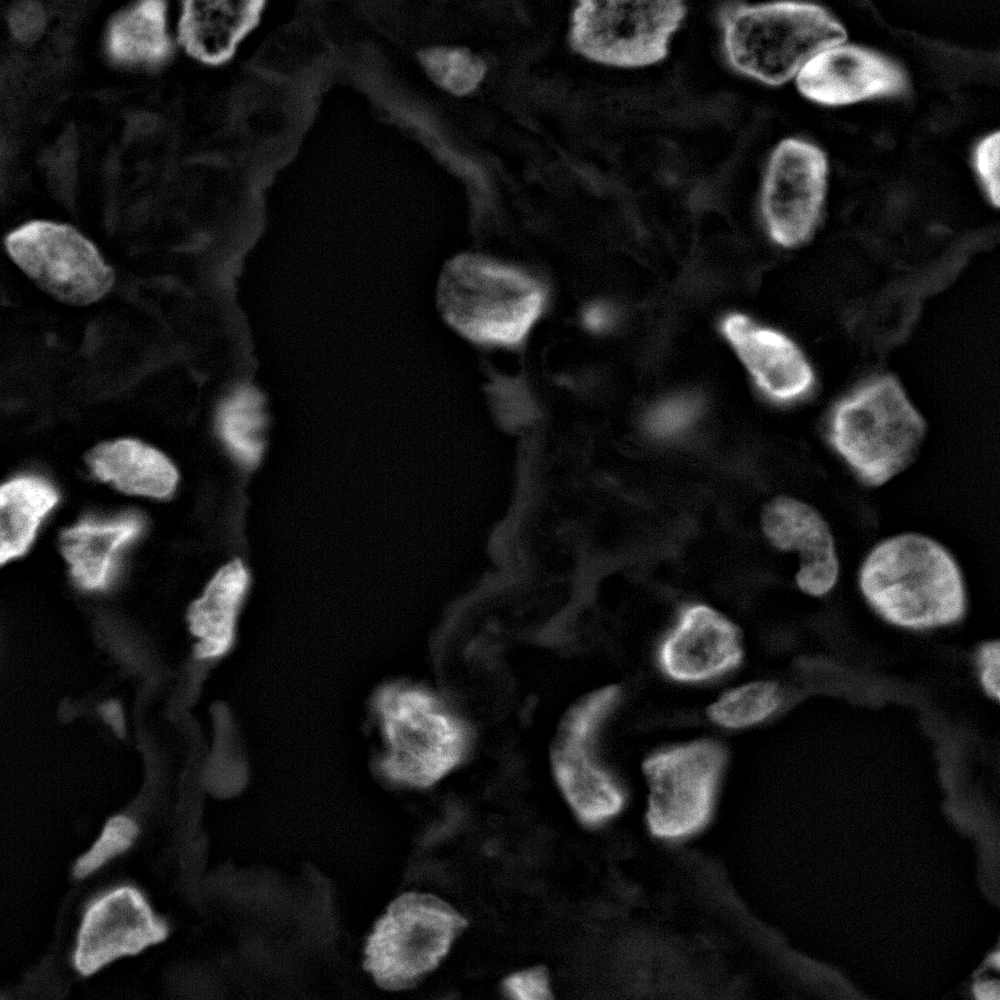

Supplement: Supplementary file 6 — Source Data [file 41467_2023_39740_MOESM6_ESM.zip › FSGS_IF/suppl_fig_8/suppl 8c pat3 region.tif]

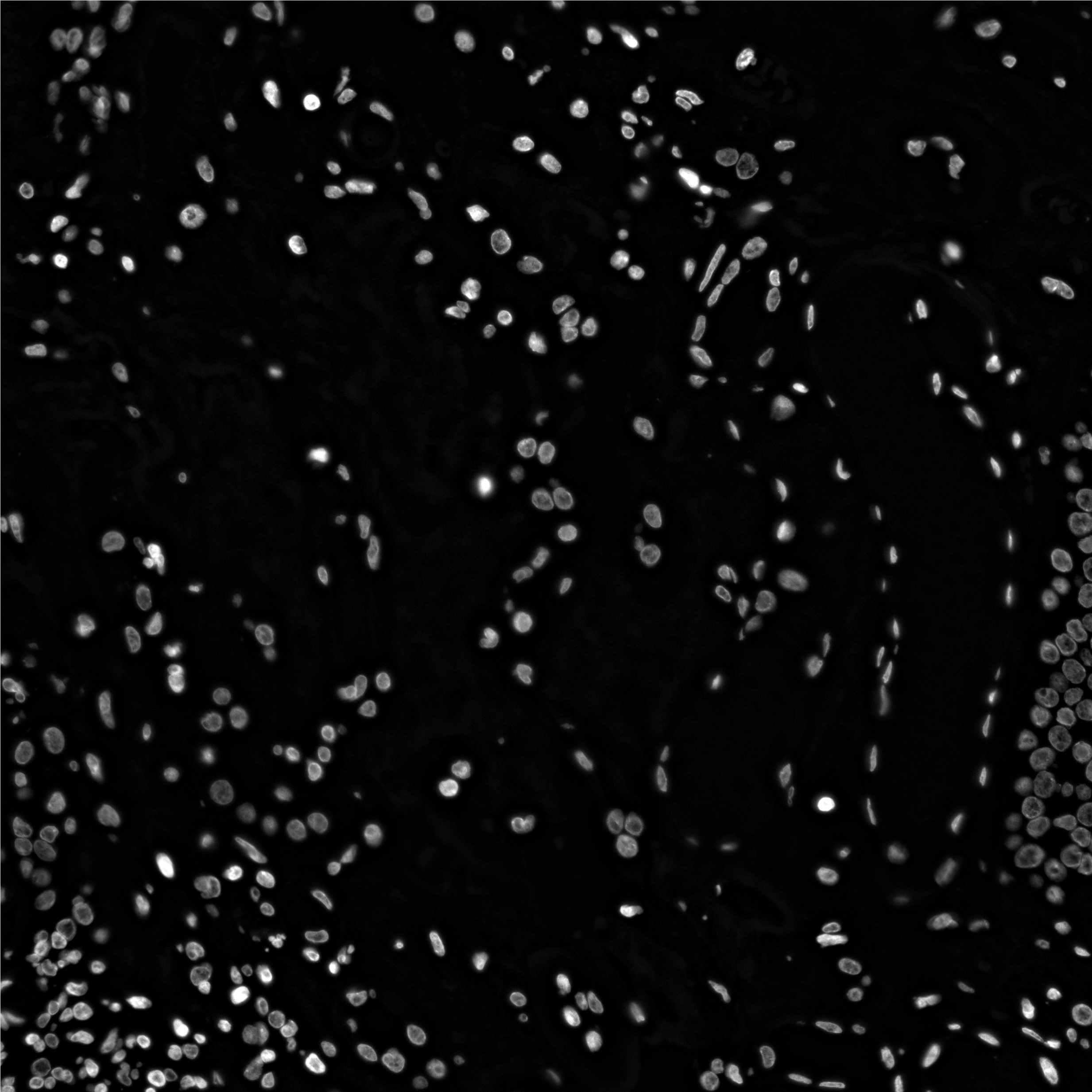

Supplement: Supplementary file 6 — Source Data [file 41467_2023_39740_MOESM6_ESM.zip › FSGS_IF/suppl_fig_8/suppl 8c pat4 overview.tif]

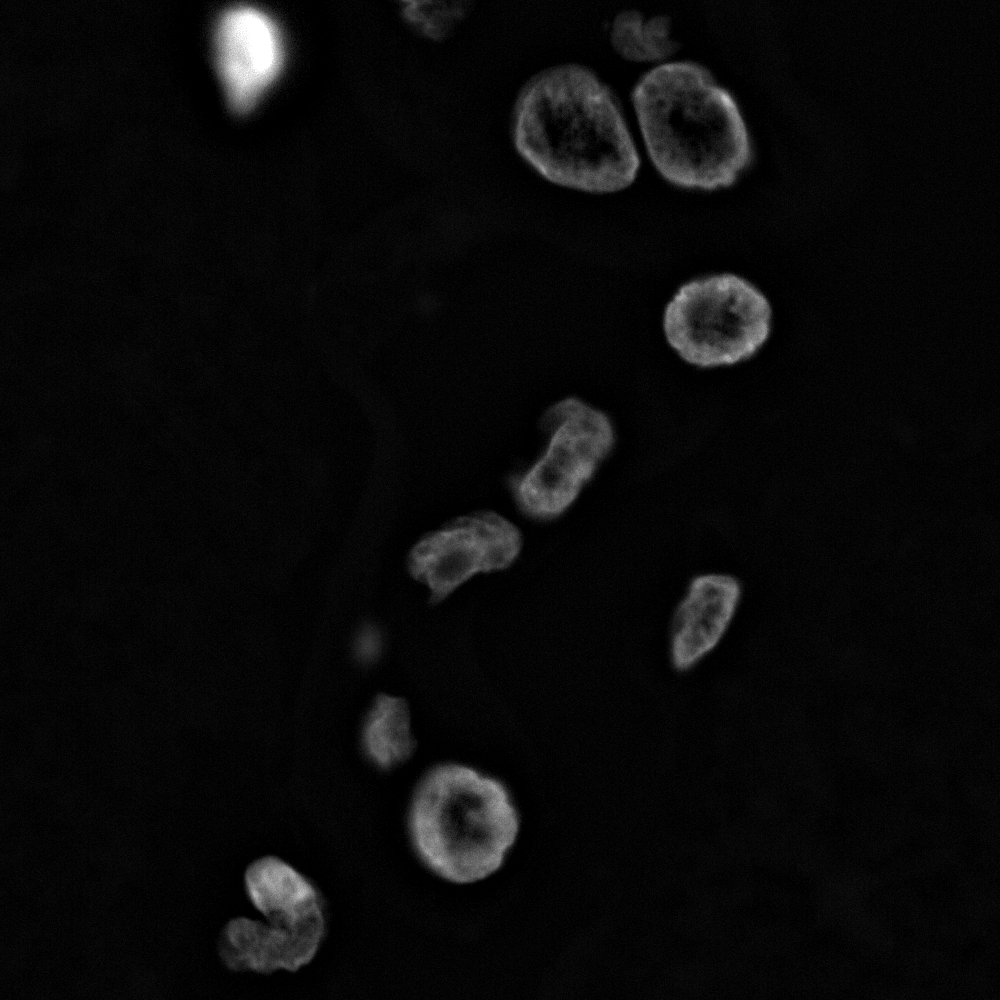

Supplement: Supplementary file 6 — Source Data [file 41467_2023_39740_MOESM6_ESM.zip › FSGS_IF/suppl_fig_8/suppl 8c pat4 region.tif]

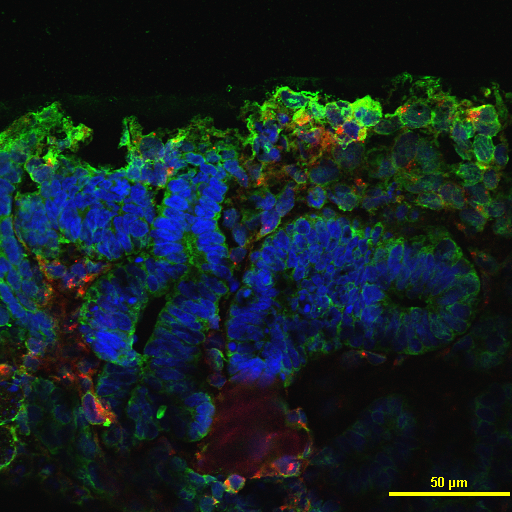

Supplement: Supplementary file 6 — Source Data [file 41467_2023_39740_MOESM6_ESM.zip › Organoid_IF/Figure 1b/ACTA2-PDGFRA-DAPI_D21.tif]

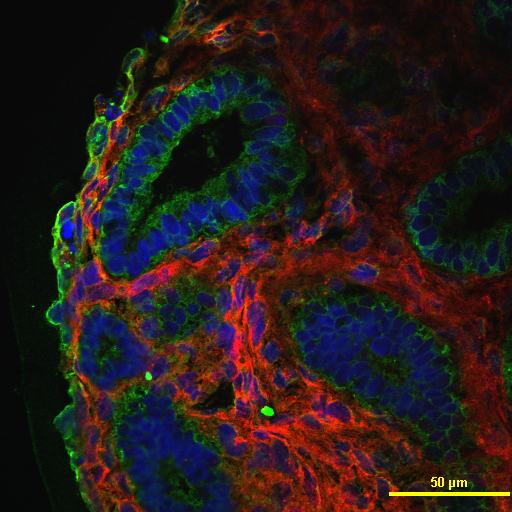

Supplement: Supplementary file 6 — Source Data [file 41467_2023_39740_MOESM6_ESM.zip › Organoid_IF/Figure 1b/ACTA2-PDGFRA-DAPI_D29.tif]

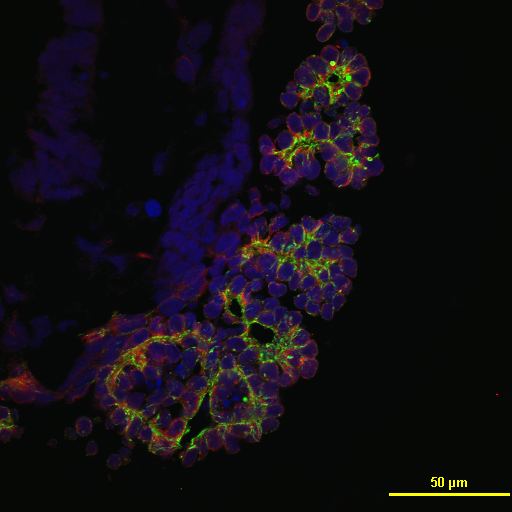

Supplement: Supplementary file 6 — Source Data [file 41467_2023_39740_MOESM6_ESM.zip › Organoid_IF/Figure 1b/SYNPO-NPHS1-DAPI_D21.tif]

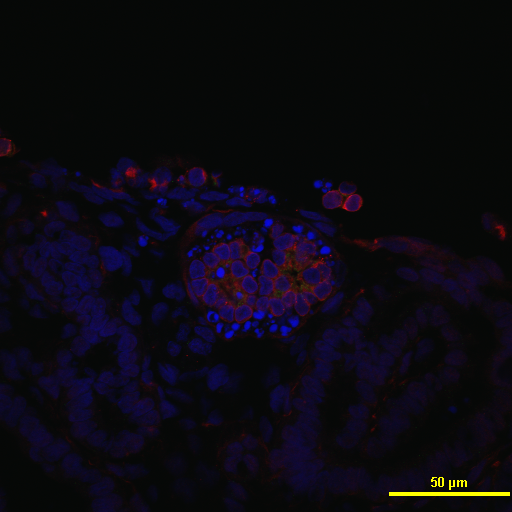

Supplement: Supplementary file 6 — Source Data [file 41467_2023_39740_MOESM6_ESM.zip › Organoid_IF/Figure 1b/SYNPO-NPHS1-DAPI_D29.tif]

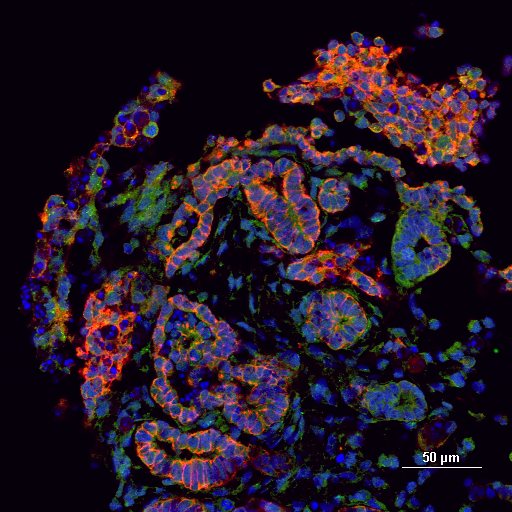

Supplement: Supplementary file 6 — Source Data [file 41467_2023_39740_MOESM6_ESM.zip › Organoid_IF/Figure 4c/CDH2-TNFRSF1A-DAPI/Merge.tif]

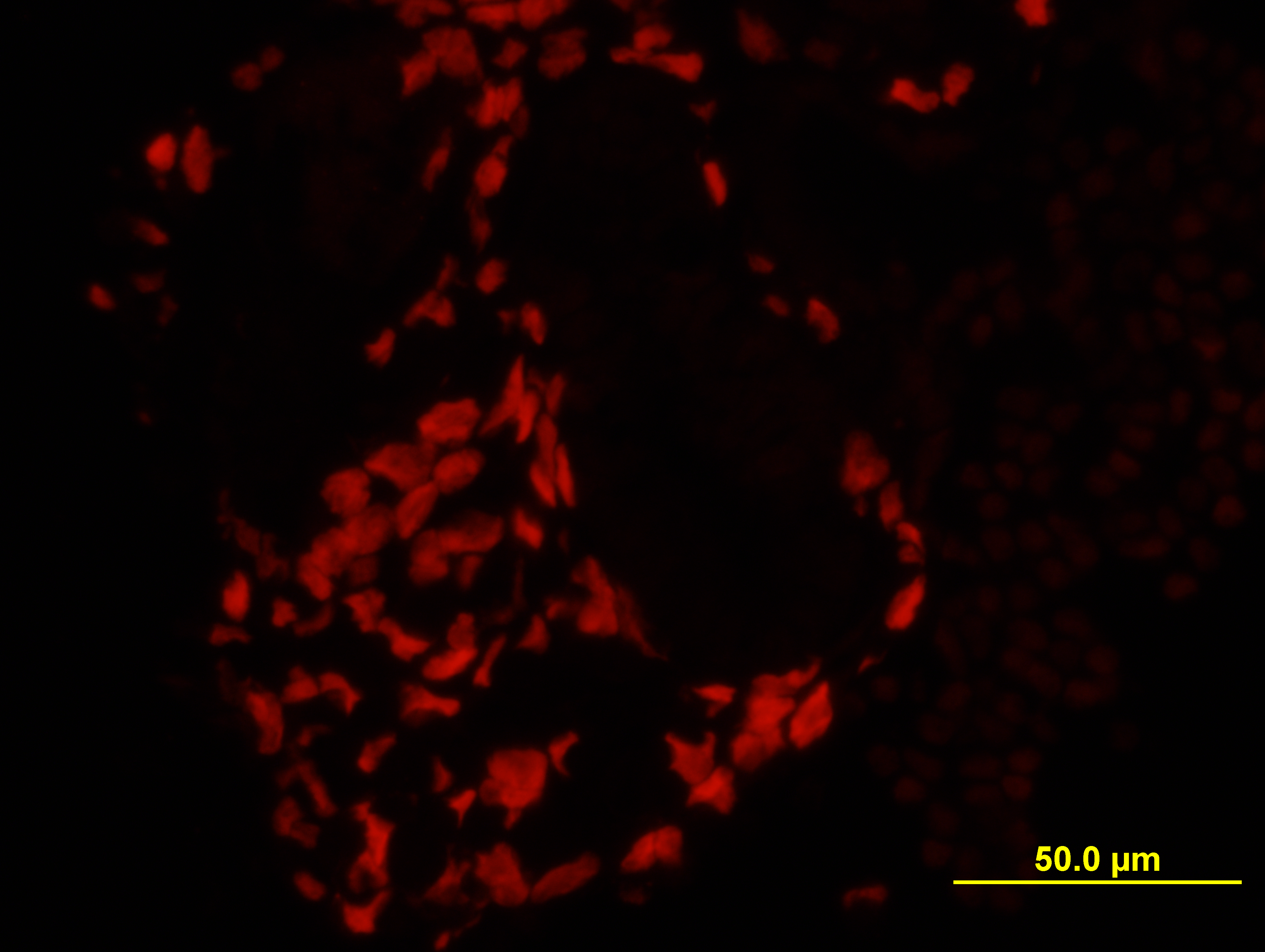

Supplement: Supplementary file 6 — Source Data [file 41467_2023_39740_MOESM6_ESM.zip › Organoid_IF/Figure 4c/MEIS-TNFRSF1A-DAPI/MEIS.tif]

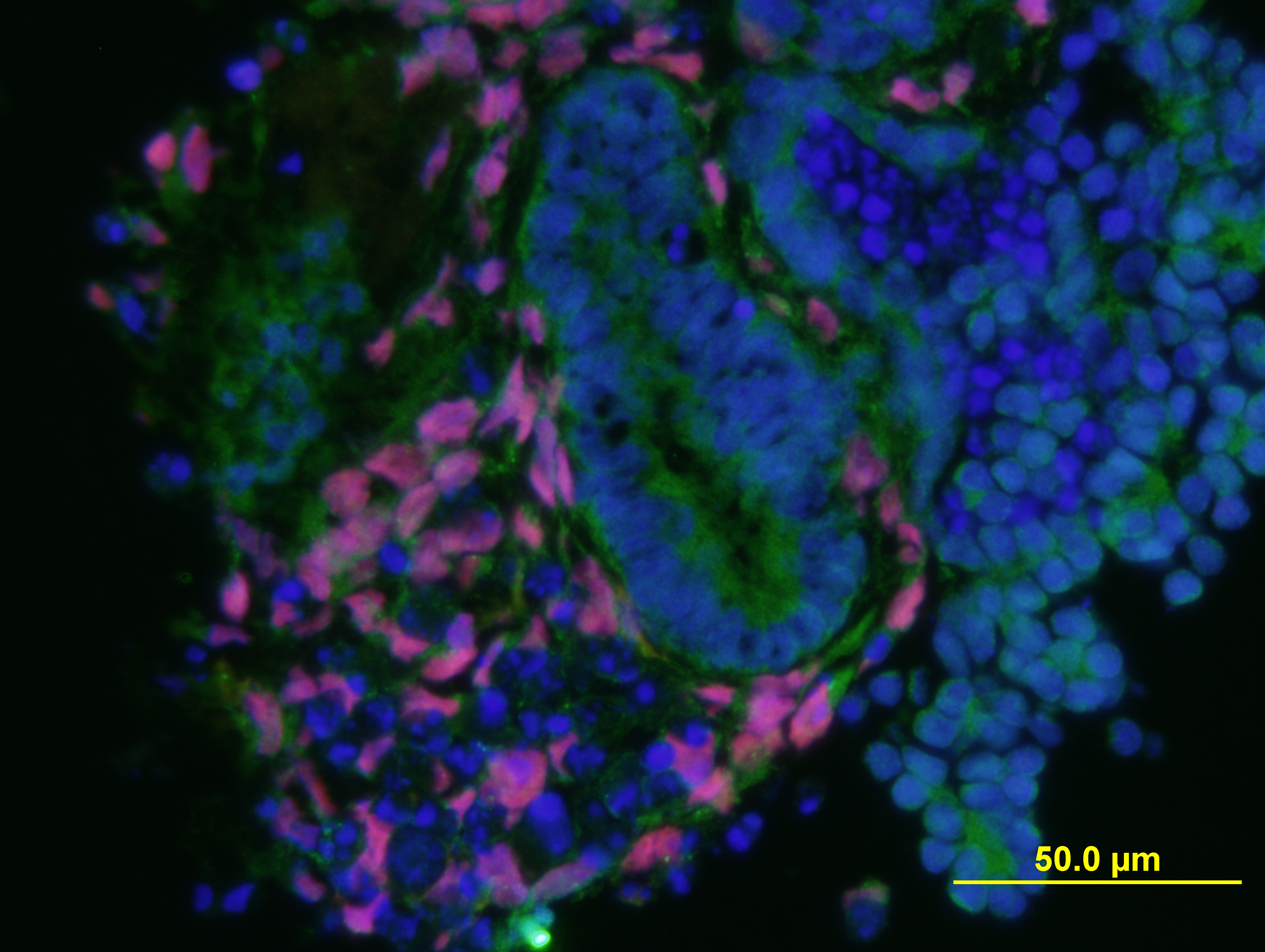

Supplement: Supplementary file 6 — Source Data [file 41467_2023_39740_MOESM6_ESM.zip › Organoid_IF/Figure 4c/MEIS-TNFRSF1A-DAPI/Merge.tif]

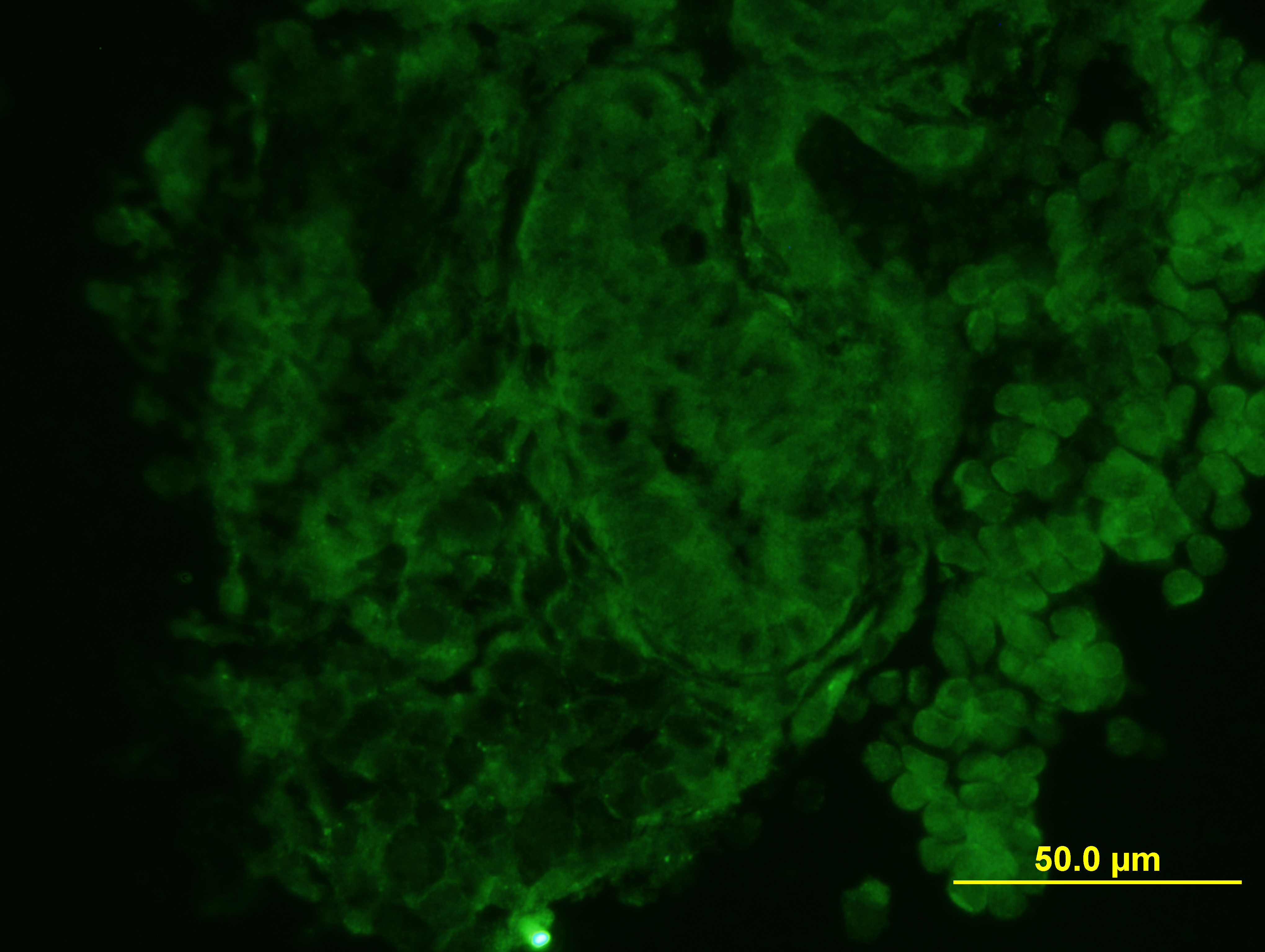

Supplement: Supplementary file 6 — Source Data [file 41467_2023_39740_MOESM6_ESM.zip › Organoid_IF/Figure 4c/MEIS-TNFRSF1A-DAPI/TNFRSF1A.tif]

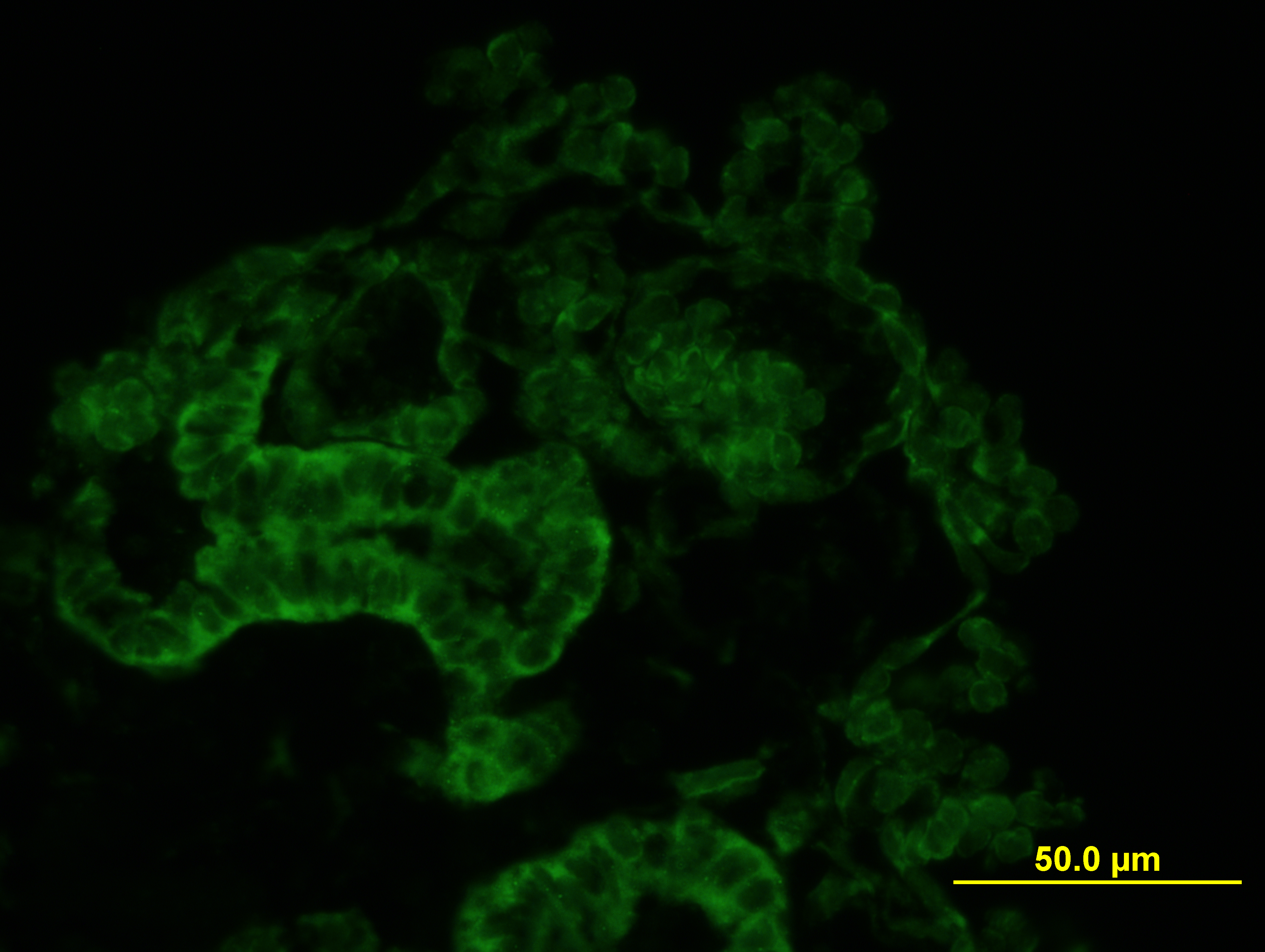

Supplement: Supplementary file 6 — Source Data [file 41467_2023_39740_MOESM6_ESM.zip › Organoid_IF/Suppl. Fig. 4a/C3-SYNPO-DAPI/C3.tif]

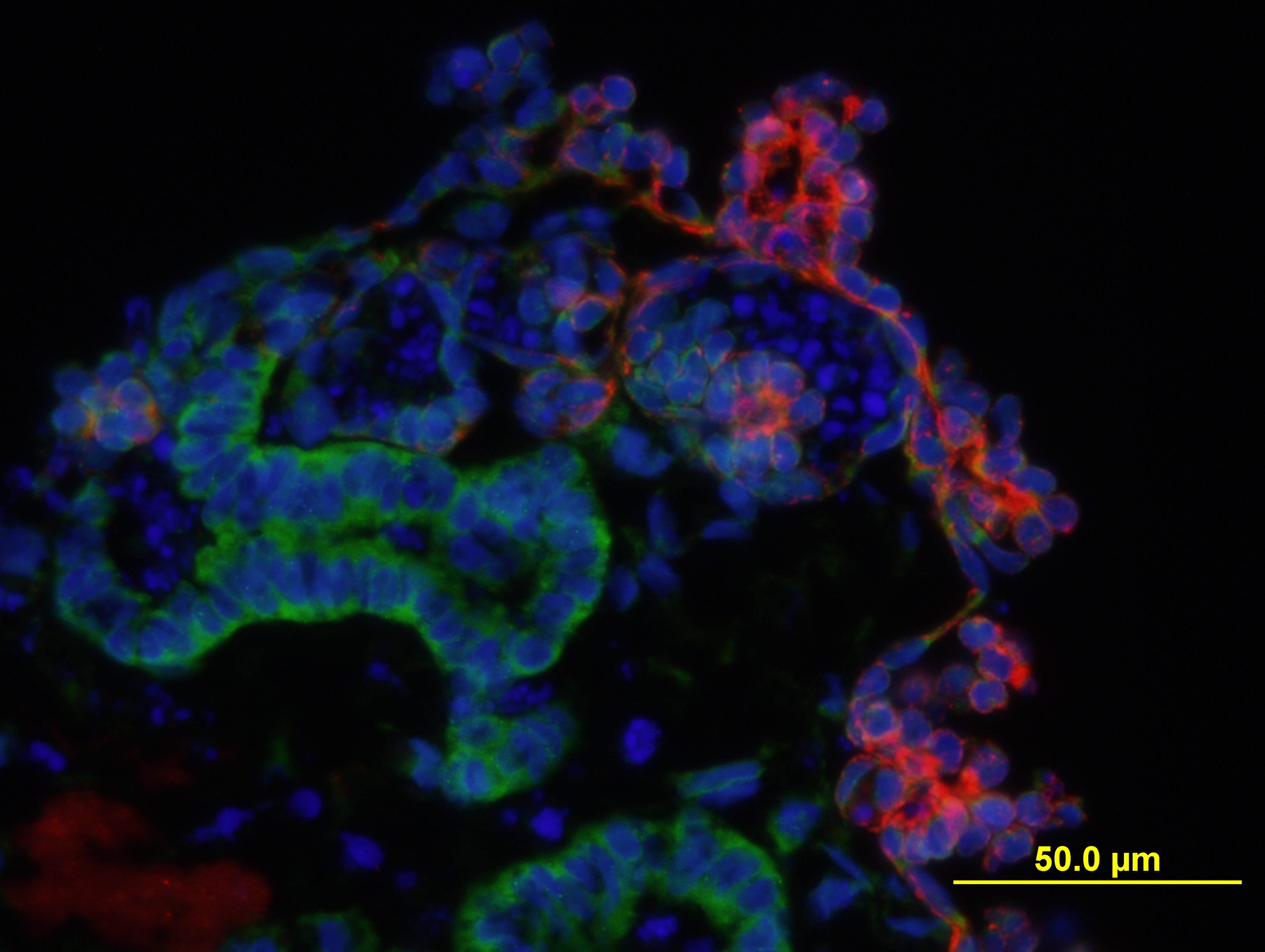

Supplement: Supplementary file 6 — Source Data [file 41467_2023_39740_MOESM6_ESM.zip › Organoid_IF/Suppl. Fig. 4a/C3-SYNPO-DAPI/C3-SYNPO Merge.tif]

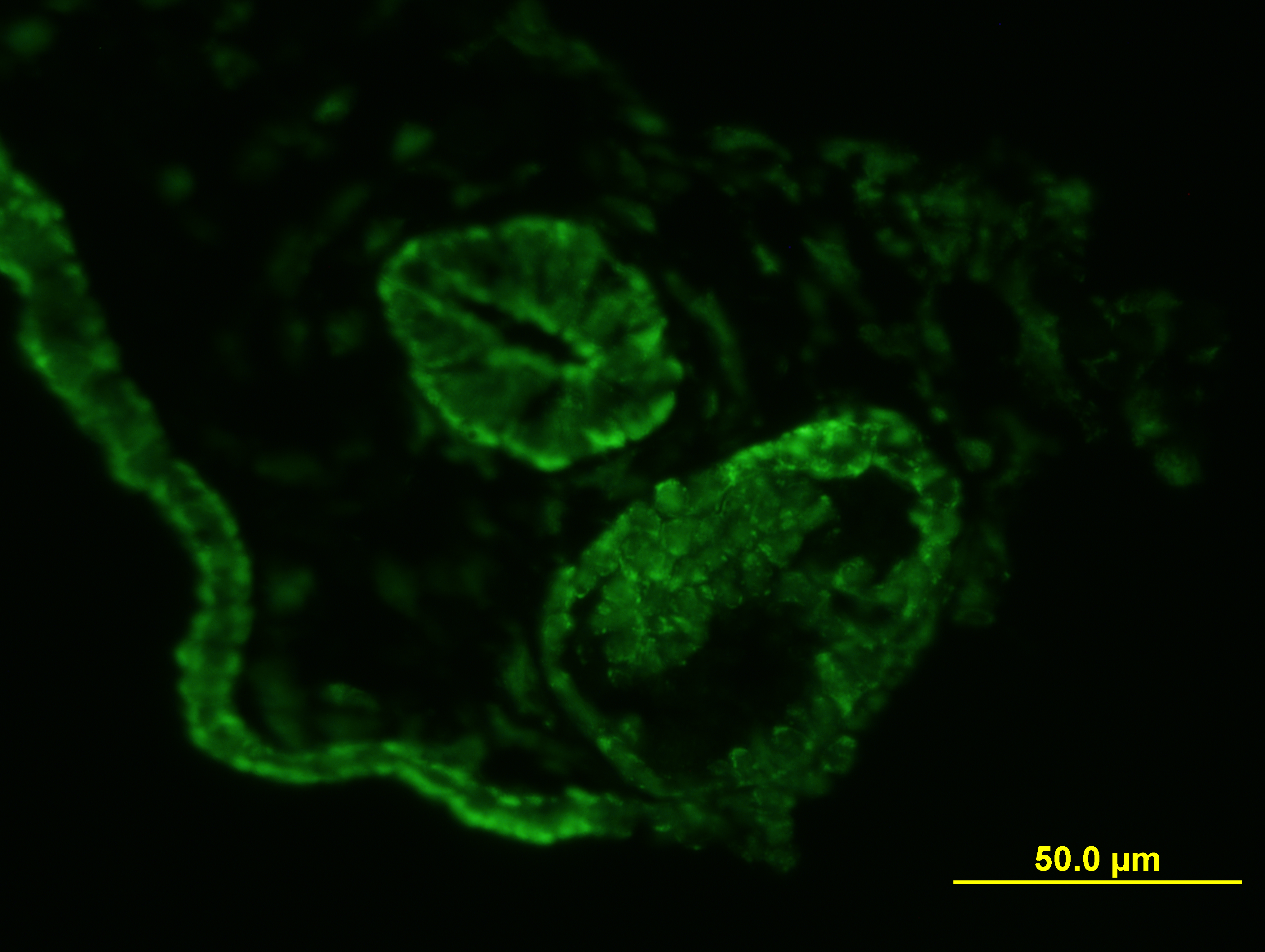

Supplement: Supplementary file 6 — Source Data [file 41467_2023_39740_MOESM6_ESM.zip › Organoid_IF/Suppl. Fig. 4a/ITGA3-SYNPO-DAPI/ITGA3.tif]

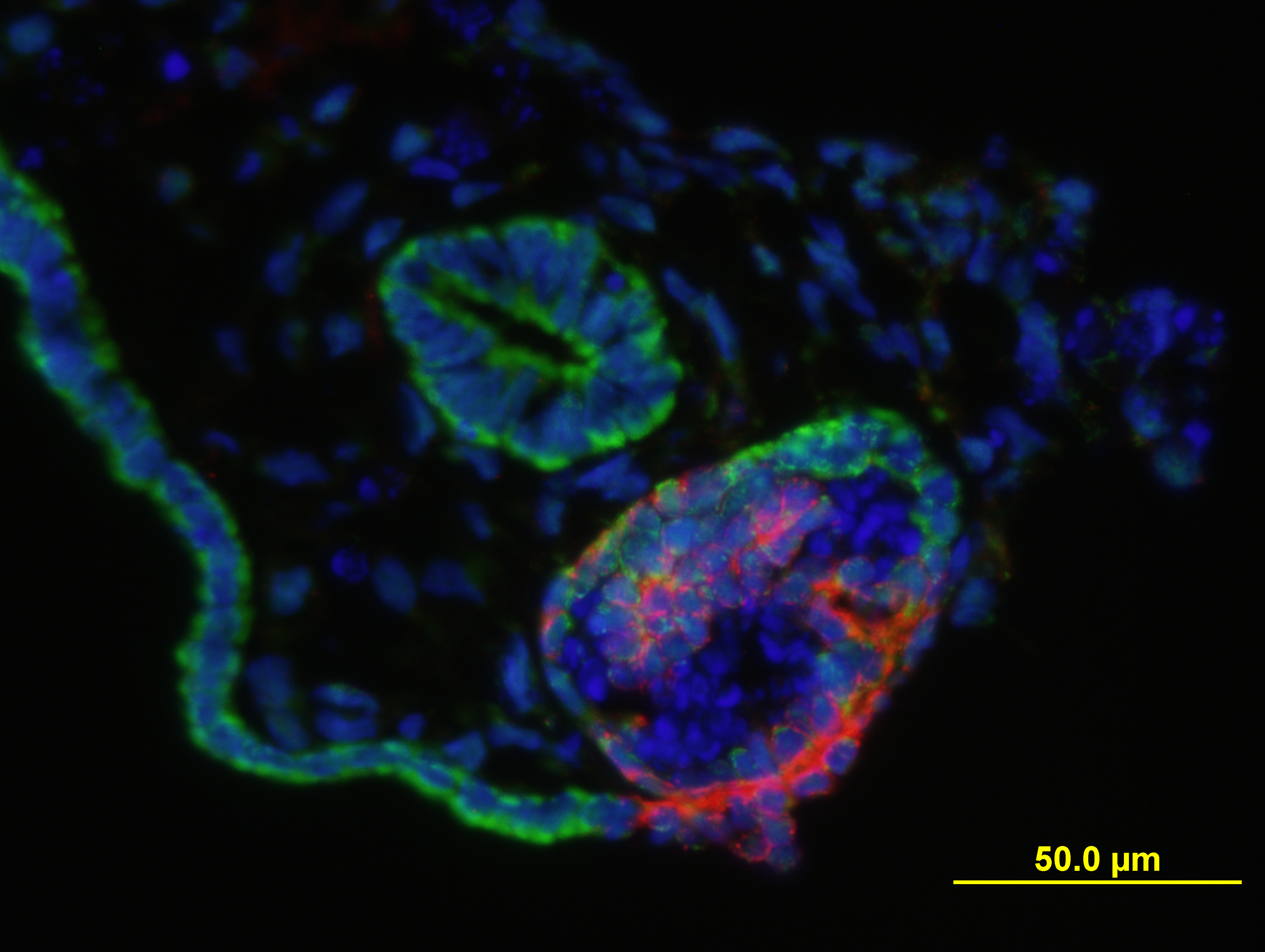

Supplement: Supplementary file 6 — Source Data [file 41467_2023_39740_MOESM6_ESM.zip › Organoid_IF/Suppl. Fig. 4a/ITGA3-SYNPO-DAPI/ITGA3-SYNPO Merge.tif]

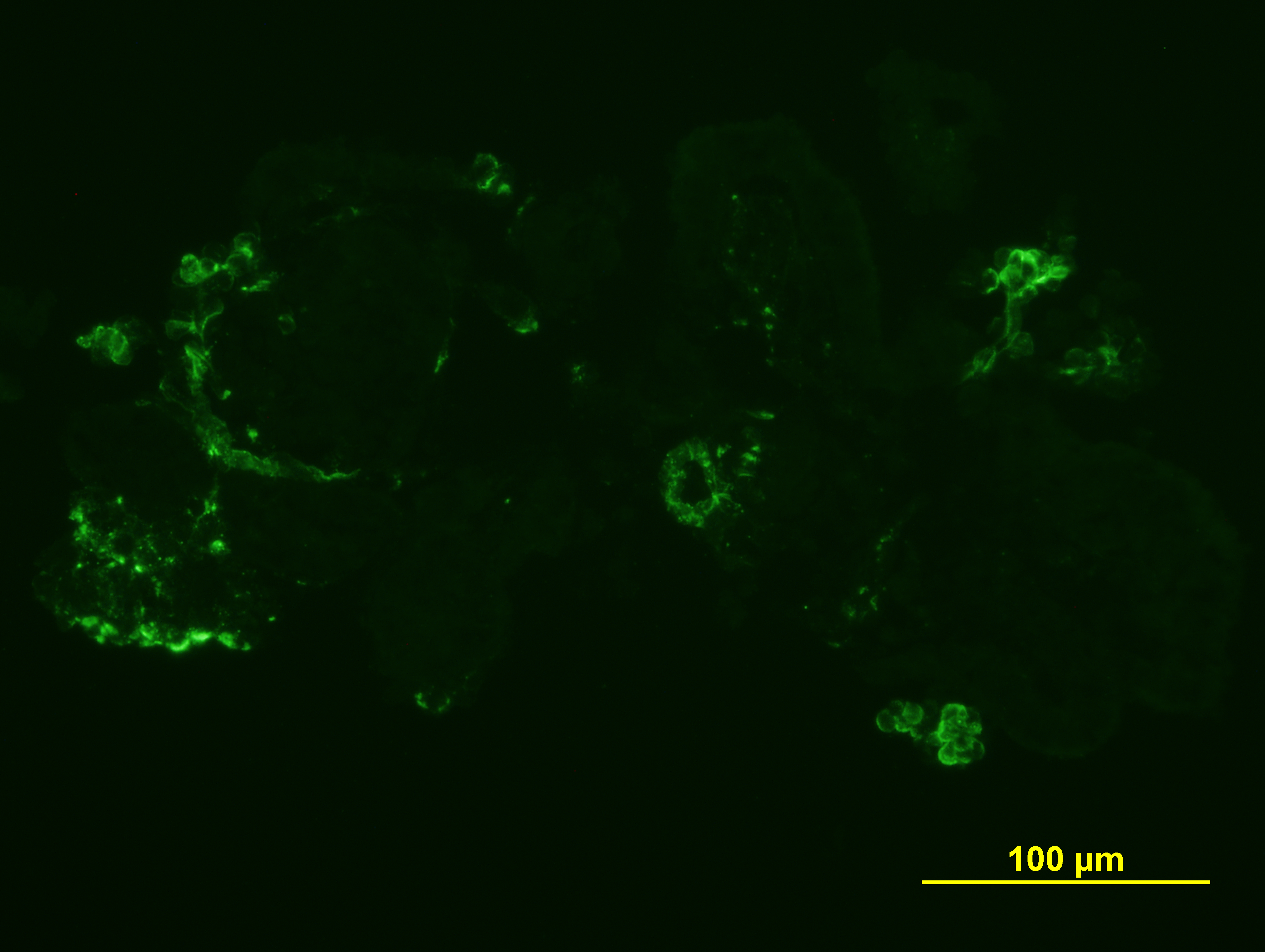

Supplement: Supplementary file 6 — Source Data [file 41467_2023_39740_MOESM6_ESM.zip › Organoid_IF/Suppl. Fig. 4a/VCAM1-PODXL-DAPI/VCAM1.tif]

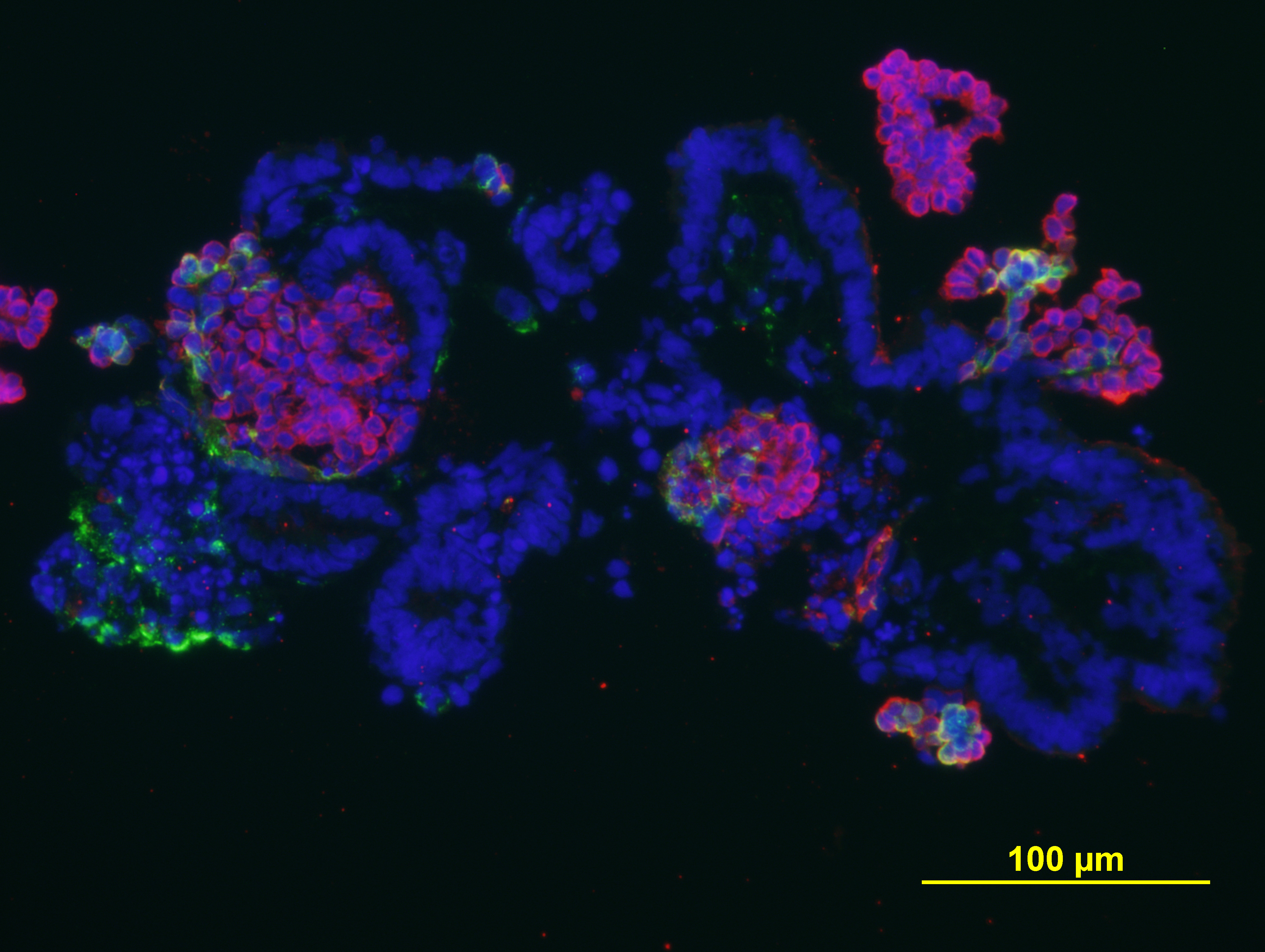

Supplement: Supplementary file 6 — Source Data [file 41467_2023_39740_MOESM6_ESM.zip › Organoid_IF/Suppl. Fig. 4a/VCAM1-PODXL-DAPI/VCAM1-PODXL Merge.tif]

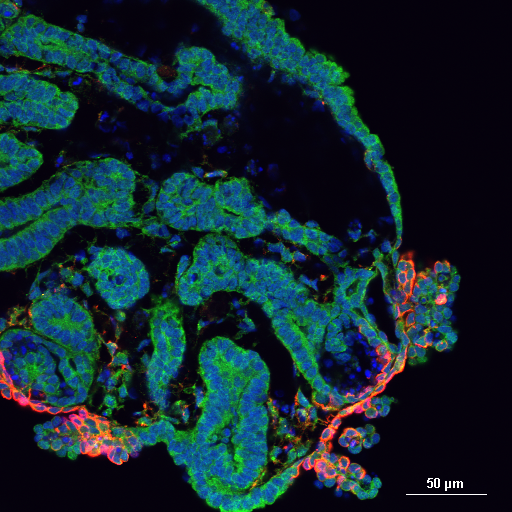

Supplement: Supplementary file 6 — Source Data [file 41467_2023_39740_MOESM6_ESM.zip › Organoid_IF/Suppl. Fig. 4b/Merge.tif]

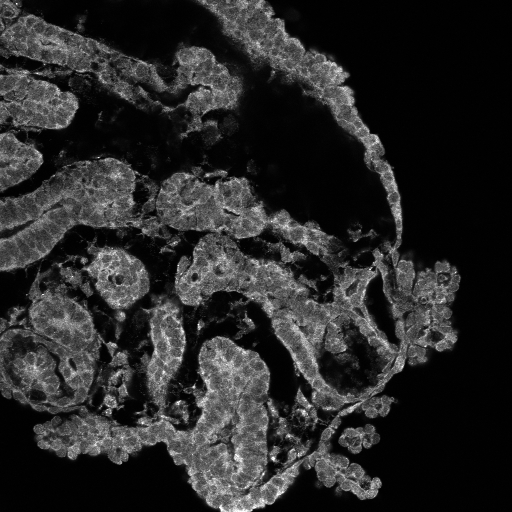

Supplement: Supplementary file 6 — Source Data [file 41467_2023_39740_MOESM6_ESM.zip › Organoid_IF/Suppl. Fig. 4b/TNFRSF1A.tif]

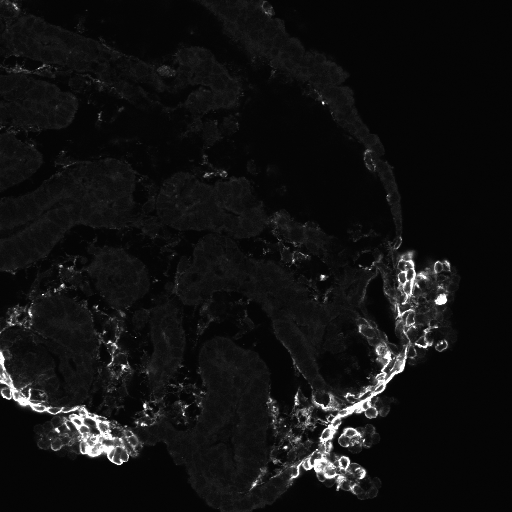

Supplement: Supplementary file 6 — Source Data [file 41467_2023_39740_MOESM6_ESM.zip › Organoid_IF/Suppl. Fig. 4b/VCAM1.tif]

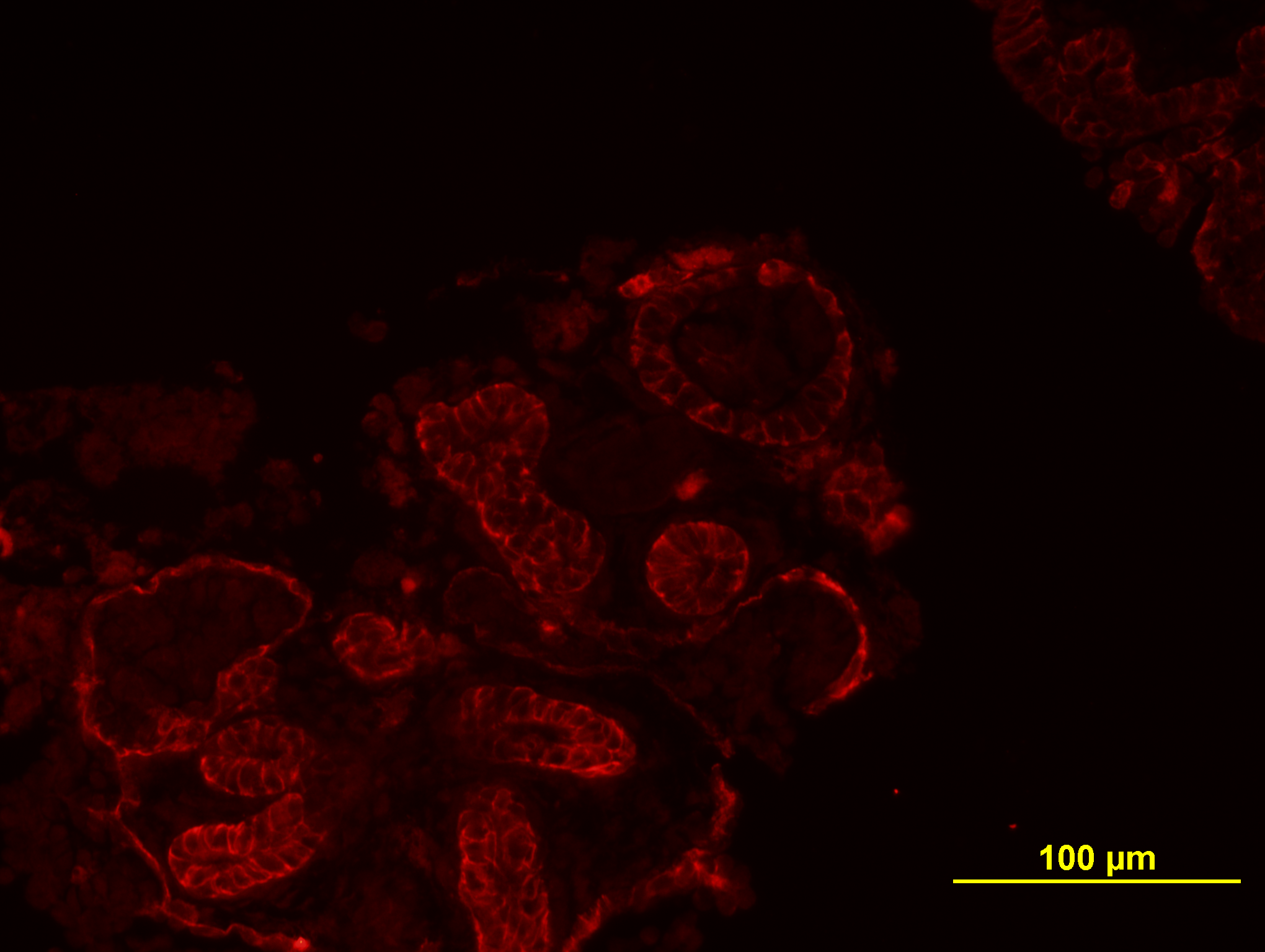

Supplement: Supplementary file 6 — Source Data [file 41467_2023_39740_MOESM6_ESM.zip › Organoid_IF/Suppl. Fig. 7a/CDH6.tif]

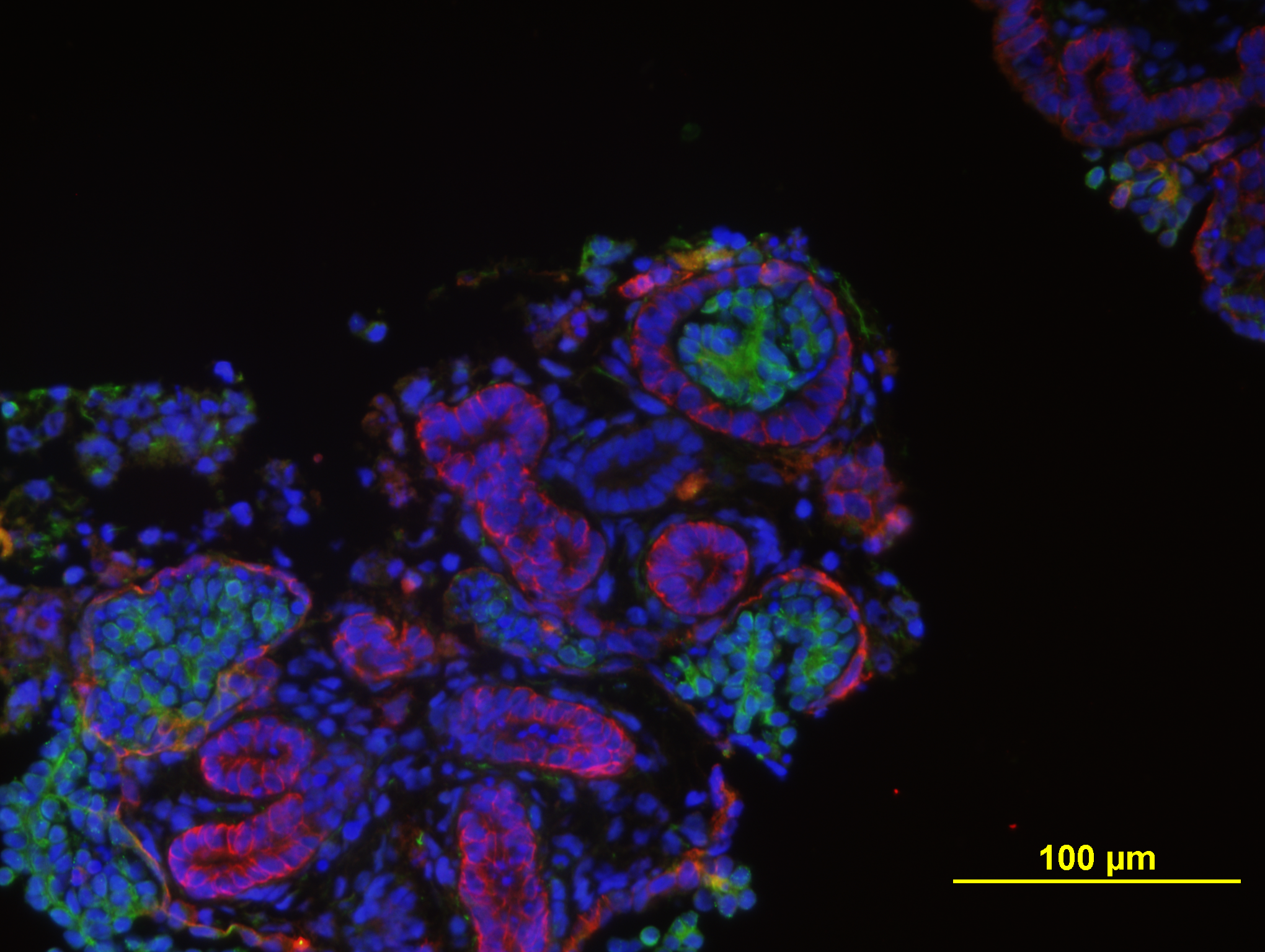

Supplement: Supplementary file 6 — Source Data [file 41467_2023_39740_MOESM6_ESM.zip › Organoid_IF/Suppl. Fig. 7a/Merge.tif]

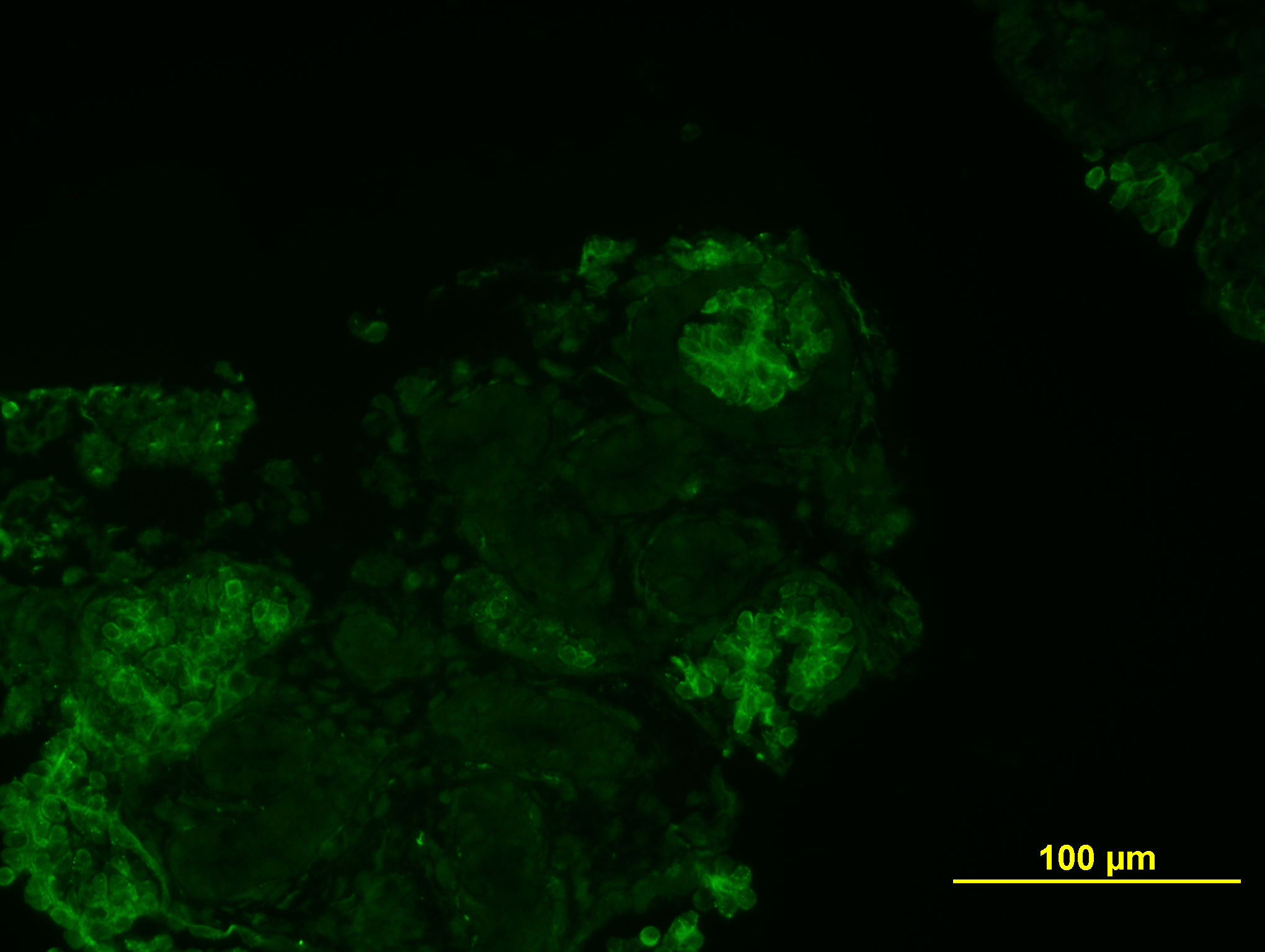

Supplement: Supplementary file 6 — Source Data [file 41467_2023_39740_MOESM6_ESM.zip › Organoid_IF/Suppl. Fig. 7a/NPHS1.tif]
